# Associations of a nutrition–inflammation index and vascular aging with mortality in adults with diabetes or prediabetes and cardiovascular disease

**DOI:** 10.1186/s12872-026-06366-8

**Published:** 2026-07-28

**Authors:** Xicheng Zhang, Jiajie Guo, Feng Luo, Danhua Zheng, Yuqing Zhang, Tianbao Chen

**Affiliations:** 1 Department of Cardiology, Quanzhou First Hospital Affiliated to Fujian Medical University, Quanzhou, 362000 China; 2 The Second School of Clinical Medicine, Guangzhou Medical University, Guangzhou, 511436 China; 3 Guizhou University of Traditional Chinese Medicine, Guiyang, 550025 China; 4 Department of Rheumatology and Immunology, The Second Affiliated Hospital of Guizhou, University of Traditional Chinese Medicine, Guiyang, 550003 China; 5 Department of Emergency Medicine, Shanghai Sixth People’s Hospital, Luoshan Section, Quanzhou, 362200 China; 6 Department of Cardiology, The First Affiliated Hospital of Wannan Medical College, Wuhu, Anhui 241001 China

**Keywords:** ALI, ePWV, Inflammation–nutrition index, Mortality, Premature death, Dysglycemia, Cardiovascular disease

## Abstract

**Background:**

Adults with prediabetes or diabetes and cardiovascular disease (CVD) face a substantial burden of premature mortality. Chronic inflammation, malnutrition and vascular aging are key pathways linking dysglycaemia and CVD to adverse outcomes. The Advanced Lung Cancer Inflammation Index (ALI) reflects systemic inflammation and nutritional status, while estimated pulse wave velocity (ePWV) serves as a surrogate of vascular aging. However, their independent and joint associations with mortality in dysglycemic adults with CVD remain unclear.

**Methods:**

NHANES 1999–2018 data comprising 101,316 participants were analyzed. After exclusions, adults ≥ 20 years with prediabetes/diabetes and CVD were included (*n* = 1,992). ALI and ePWV were analysed as continuous variables and cross-classified using maximally selected cut-points. Survey-weighted Cox models, restricted cubic splines and subgroup/sensitivity analyses were applied. Time-dependent receiver operating characteristic curves and C-indices were used to compare prognostic discrimination.

**Results:**

Higher ALI was inversely associated with all-cause, cardiovascular, and premature mortality, whereas higher ePWV was positively associated with all-cause and cardiovascular mortality. Per 10-unit increment in ALI, fully adjusted hazard ratios (HRs) were 0.899 (95% CI 0.872–0.928) for all-cause mortality, 0.938 (0.885–0.995) for cardiovascular mortality, and 0.889 (0.837–0.945) for premature mortality. For ePWV, the corresponding HRs per 1 m/s increment were 1.139 (1.072–1.211) for all-cause mortality and 1.216 (1.102–1.341) for cardiovascular mortality. In joint analyses, combined ALI–ePWV categories showed a risk gradient: participants with high ALI/low ePWV had the lowest risks of all-cause mortality (HR 0.382, 95% CI 0.280–0.520), cardiovascular mortality (HR 0.379, 95% CI 0.228–0.630), and premature mortality (HR 0.402, 95% CI 0.245–0.659) compared with those with low ALI/high ePWV.

**Conclusions:**

ALI and ePWV provide complementary information on systemic inflammation–nutrition status and vascular ageing, and their combined categories define distinct risk strata for mortality in adults with dysglycaemia and CVD. Their combined categories may help characterize mortality-risk heterogeneity and inform future risk-assessment studies in this high-risk population.

**Supplementary Information:**

The online version contains supplementary material available at https://doi.org/10.1186/s12872-026-06366-8.

## Background

 In adults with diabetes, cardiovascular disease (CVD) remains a major contributor to mortality despite contemporary multifactorial risk control and cardioprotective therapy [1, 2]. Prediabetes and diabetes are part of a broader cardiometabolic risk continuum in which insulin resistance, hyperglycemia, dyslipidemia, obesity, inflammation, and vascular dysfunction frequently coexist and reinforce each other [2, 3]. Among individuals already living with CVD, the coexistence of prediabetes or diabetes identifies a particularly vulnerable secondary-prevention population with substantial residual risk for long-term and premature mortality. Therefore, markers that capture complementary dimensions of inflammation–nutrition status and vascular aging may help characterize mortality risk and identify higher-risk profiles in this population.

The advanced lung cancer inflammation index (ALI) was developed to assess systemic inflammation and nutritional status in metastatic non-small cell lung cancer, with lower ALI linked to poorer survival [4]. Beyond oncology, emerging evidence links ALI to cardiovascular risk: higher ALI is linked to lower all-cause and cardiovascular mortality in older adults with heart failure, hypertensive cohorts, and broader cardiometabolic populations [5–7]. Recent National Health and Nutrition Examination Survey (NHANES)-based analyses further suggest that ALI tracks long-term mortality in community subgroups (for example, sarcopenic adults), underscoring its potential as a pragmatic inflammation–nutrition composite for population research [8]. The ALI incorporates body-size- and adiposity-related information through BMI, and its individual components are independently associated with cardiovascular prognosis: lower serum albumin predicts increased mortality, whereas an elevated neutrophil-to-lymphocyte ratio (NLR) correlates with both higher mortality and a greater risk of cardiovascular events [5, 9].

Estimated pulse wave velocity (ePWV), a validated surrogate for carotid–femoral PWV, is calculated from chronological age and mean arterial pressure [10, 11]. ePWV captures vascular aging and has consistently predicted mortality across settings, including the SPRINT trial, the general US population, and diabetes cohorts from NHANES [12–14]. In addition, reviews and meta-analyses indicate that lipid abnormalities are associated with higher PWV and that statins can modestly lower arterial stiffness, further situating ePWV within a risk pathway that intersects with lipid biology [15].

Although ALI and ePWV have each been associated with adverse outcomes, they have largely been evaluated as separate markers. This leaves an important gap because nutrition–inflammation status and vascular aging may represent different but intersecting dimensions of cardiometabolic risk. Hyperglycemia and advanced glycation end-products can promote collagen cross-linking and extracellular matrix remodeling, thereby reducing arterial and myocardial compliance and accelerating vascular aging [16]. Insulin resistance may further contribute to oxidative stress, endothelial dysfunction, altered lipid metabolism, and chronic low-grade inflammation, all of which are relevant to atherosclerosis and adverse cardiovascular outcomes [3, 17]. Obesity and adiposity-related metabolic disturbance may amplify these processes, partly through insulin resistance and inflammatory activation [18]. Accordingly, in adults with both dysglycemia and established CVD, mortality risk may reflect the convergence of nutrition–inflammation status and vascular aging rather than either domain alone.

However, whether the joint ALI–ePWV profile is associated with mortality risk in adults with prediabetes or diabetes and established CVD remains insufficiently characterized, particularly for premature mortality. Using mortality-linked NHANES data, we examined the individual and joint associations of ALI and ePWV with all-cause, cardiovascular, and premature mortality (< 75 years) in U.S. adults with prediabetes or diabetes and co-existing CVD. We hypothesized that participants with concurrent low ALI (indicating worse systemic inflammation–nutrition status) and high ePWV (indicating greater arterial stiffness) would have the highest risks of all three endpoints, whereas high ALI combined with low ePWV would identify a lower-risk phenotype. This population-based analysis examined the joint ALI–ePWV profile as an integrated nutrition–inflammation and vascular-aging approach to characterizing mortality risk in this high-risk secondary-prevention population.

## Materials and methods

### Study population

NHANES, an ongoing survey representative of the U.S. civilian, non-institutionalized population, evaluates health and nutrition biennially using interviews, physical exams, and laboratory analyses [19].

Data from NHANES 1999–2018 (*N* = 101,316) were pooled for analysis. Participants were excluded if they were younger than 20 years (*n* = 46,235) or pregnant at the time of the survey (*n* = 1,547), leaving 53,534 individuals. We further excluded participants without linked mortality follow-up (*n* = 2,732), those without diabetes or prediabetes (*n* = 38,312), and those without CVD (*n* = 9,671), yielding 2,819 eligible participants.

Prediabetes and diabetes were defined according to the American Diabetes Association (ADA) diagnostic framework using available self-reported diagnosis, medication use, fasting plasma glucose, HbA1c, and 2-hour oral glucose tolerance test information. Oral glucose tolerance test (OGTT)-based criteria were applied only in NHANES cycles and participants with available OGTT data. CVD was defined as a self-reported physician or health-professional diagnosis of at least one of the following conditions in the NHANES Medical Conditions Questionnaire: congestive heart failure, coronary heart disease, angina, myocardial infarction, or stroke. The corresponding NHANES variables were MCQ160B–MCQ160F, and participants with a “Yes” response to any of these items were classified as having CVD. Detailed coding criteria for prediabetes, diabetes, and CVD are provided in Table S1 [20].

**Fig. 1:**
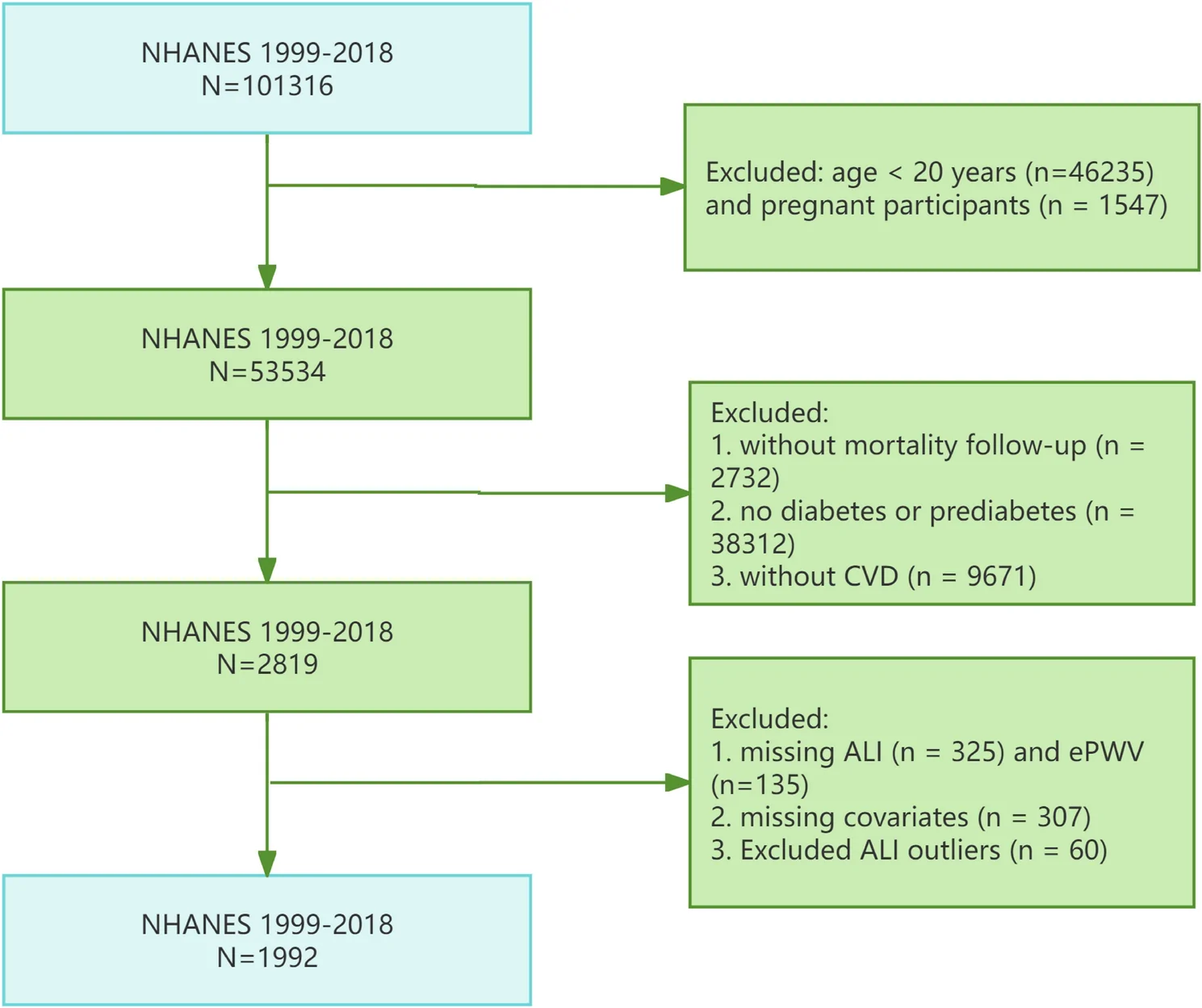
Flowchart of the study population

Additional exclusions were applied for missing ALI (*n* = 325) and missing ePWV (*n* = 135), leaving 2,359 participants with complete core exposure data. Missingness of key analytic variables was then summarized before covariate exclusions and in the final analytic cohort (Table S6). We further excluded participants with missing prespecified covariates (*n* = 307) and extreme ALI values > 150 (*n* = 60). The primary analyses were therefore conducted using a complete-case approach for the prespecified covariates, and no imputation was applied in the primary analysis. The ALI threshold of 150 was selected after distributional assessment because values above this level were sparse and clearly separated from the main exposure distribution, suggesting potential high-leverage observations from unusual combinations of BMI, albumin, and NLR. The original ALI distribution is shown in Figure S1, and sensitivity analyses including participants with ALI > 150 were performed to assess the robustness of this decision. After all exclusions, the final analytic cohort included 1,992 adults (Fig. 1).

### Outcomes

Mortality status was determined through probabilistic linkage of NHANES records to the National Death Index (NDI) by the National Center for Health Statistics (NCHS), using identifiers such as Social Security number, date of birth, and demographic information. Follow-up continued until death or December 31, 2019, whichever occurred first; participants not linked to a death record were considered alive at the end of follow-up.

All-cause mortality was defined as death from any cause. Cardiovascular mortality was classified according to International Classification of Diseases, 10th Revision (ICD-10) codes I00–I09, I11, I13, and I20–I51. Premature mortality was defined as death before age 75, in line with the average life expectancy of U.S. adults during the study period [21]. For analyses of premature mortality, participants aged 75 years or older at baseline were excluded, and follow-up was censored at the 75th birthday among those who survived beyond that age.

### ALI and ePWV

ALI was calculated as follows: $\:ALI=\frac{BMI\:\left(kg/{m}^{2}\right)*albumin\:level\:\left(g/dL\right)}{NLR}$, where the NLR was defined as: $\:NLR=\:\frac{\mathrm{N}\mathrm{e}\mathrm{u}\mathrm{t}\mathrm{r}\mathrm{o}\mathrm{p}\mathrm{h}\mathrm{i}\mathrm{l}\:\mathrm{c}\mathrm{o}\mathrm{u}\mathrm{n}\mathrm{t}}{\mathrm{L}\mathrm{y}\mathrm{m}\mathrm{p}\mathrm{h}\mathrm{o}\mathrm{c}\mathrm{y}\mathrm{t}\mathrm{e}\:\mathrm{c}\mathrm{o}\mathrm{u}\mathrm{n}\mathrm{t}}$ [4].

ePWV was calculated with age (years) and mean blood pressure (MBP, mmHg), as shown below:$$\begin{aligned} &ePWV=9.587-0.402\times\,age+4.560\times\,10^{-3}\times\,age^2-2.621\times\,10^{-5}\times\,age^2\\&\times\,MBP+3.176\times\,10^{-3}\times\,age\times\,MBP-1.832\times\,10^{-2}\times\,MBP \end{aligned}$$, where the MBP was defined as: $$\begin{aligned} &MBP=diastolic\,blood\,pressure\left(DBP\right)+0.4\\&\times\left[systolic\,blood\,pressure\left(SBP\right)-DBP\right] \end{aligned}$$. Following 5 min of quiet sitting, a trained health technician obtained three consecutive blood pressure measurements [22].

### Covariates

Covariates were chosen a priori according to clinical reasoning and in line with prior NHANES-based and cardiovascular epidemiology studies investigating arterial stiffness and cardiometabolic risk [13, 14]. The fully adjusted model included age, sex, race/ethnicity, marital status, education level, smoking status, alcohol consumption, hypertension, hyperlipidemia, cancer, Healthy Eating Index-2015 (HEI-2015), and estimated glomerular filtration rate (eGFR). Smoking status was classified as never, former, or current smoking. Alcohol consumption was classified as never, former, or current drinking. Hypertension was defined by measured blood pressure, self-reported physician diagnosis, or antihypertensive medication use. Hyperlipidemia was defined by abnormal lipid measurements or lipid-lowering medication use. Abnormal lipid measurements included total cholesterol ≥ 200 mg/dL, LDL-C ≥ 130 mg/dL, triglycerides ≥ 150 mg/dL, or HDL-C < 40 mg/dL in men and < 50 mg/dL in women. Lipid-lowering medication use was identified from the NHANES Blood Pressure and Cholesterol Questionnaire. Cancer was defined by self-reported physician diagnosis. Detailed covariate definitions, NHANES variable coding, and medication-related criteria are provided in Text S1.

Diet quality was assessed using the HEI-2015 score, with component definitions and scoring standards shown in Table S2. Kidney function was assessed using eGFR, calculated with the 2021 Chronic Kidney Disease Epidemiology Collaboration creatinine equation without race [23, 24]. In sensitivity analyses, CVD burden was defined as the number of self-reported CVD conditions and categorized as 1, 2, or ≥ 3 conditions; HbA1c was additionally evaluated as a marker of glycemic control.

### Statistical analysis

All analyses used R 4.4.1 (R Foundation); two-sided *P* < 0.05 denoted statistical significance. Analyses incorporated NHANES’s complex design using strata (SDMVSTRA), primary sampling units (SDMVPSU), and Medical Examination Center (MEC) weights; variances were estimated with Taylor series linearization, following established analytic guidance. Because we pooled 10 two-year cycles (1999–2018) and all exposures were derived from the MEC examination, multi-cycle weights were constructed as (2/10) ×WTMEC4YR for 1999–2002 and (1/10) ×WTMEC2YR for each cycle from 2003 to 2018, consistent with published practice when aggregating NHANES data across multiple cycles.

Baseline characteristics were summarized across ALI quartiles as survey-weighted means with standard errors (SE) for continuous variables and unweighted counts with survey-weighted percentages for categorical variables. Differences across quartiles were assessed using design-based Wald tests for continuous variables and Rao–Scott adjusted chi-square tests for categorical variables. ALI and ePWV were analyzed as continuous variables and quartiles. Maximally selected rank statistics were used to determine exploratory cut points of 34.33 for ALI and 10.18 m/s for ePWV (Figure S2), which were then used to construct four joint ALI–ePWV categories. Survey-weighted Cox proportional hazards models were used to estimate hazard ratios (HRs) and 95% confidence intervals (CIs) for the associations of ALI, ePWV, and joint ALI–ePWV categories with all-cause, cardiovascular, and premature mortality. Three models were fitted: Model 1 was unadjusted; Model 2 adjusted for age, sex, and race/ethnicity; and Model 3 further adjusted for marital status, education level, smoking status, alcohol consumption, hypertension, hyperlipidemia, cancer, HEI-2015, and eGFR. Although ePWV is calculated from age and mean blood pressure, age and hypertension were retained in the primary fully adjusted model because they are major mortality-related clinical covariates and established cardiovascular risk factors in adults with diabetes and CVD [1, 2]. Collinearity diagnostics and alternative ePWV adjustment models excluding hypertension, age, or both were performed to evaluate the robustness of the ePWV-related associations. For premature mortality, analyses were restricted to participants younger than 75 years at baseline, and follow-up was censored at the 75th birthday among participants who survived beyond that age. Events per exposure category were reported in the Cox regression tables. Kaplan–Meier curves with log-rank tests were used to describe survival differences across ALI quartiles, ePWV quartiles, and joint ALI–ePWV categories. To explore dose–response shapes and potential departures from linearity, we fitted restricted cubic spline (RCS) terms with three knots at the 10th, 50th, and 90th percentiles for ALI and ePWV in fully adjusted Cox models and tested nonlinearity; when indicated, we examined threshold effects using two-piecewise (segmented) Cox models with data-driven breakpoints. Time-dependent receiver operating characteristic (ROC) curves and areas under the curve (AUCs) were estimated at 5, 10, and 15 years. Optimal cutoffs were selected by maximizing the Youden index, as used in previous ROC-based studies [25, 26]. Detailed ROC metrics, including cutoff, Youden index, sensitivity, specificity, positive predictive value, and negative predictive value, were summarized in Table S4. Harrell’s time-dependent concordance index (C-index) curves were further used to compare discrimination across follow-up. Effect modification was evaluated by stratifying the primary Cox models according to age (< 70 vs. ≥ 70 years) and sex, with multiplicative interaction terms included to test for statistical interaction.

Proportional hazards assumptions for the Cox models were evaluated using Schoenfeld residual-based tests, including global tests and variable-specific tests for the core exposure term and each covariate in the fully adjusted Model 3; detailed diagnostic results are provided in Table S11. Sensitivity analyses included additional adjustment for CVD burden and HbA1c, analyses including participants with ALI > 150, exclusion of deaths occurring within the first two years of follow-up, collinearity diagnostics and alternative ePWV adjustment sets, accelerated failure time models for potential proportional-hazards violations, and Fine–Gray competing-risk models for cardiovascular mortality treating non-cardiovascular death as a competing event. Fasting glucose was not included in additional-adjustment models because it was available only in the fasting subsample and had substantial missingness across pooled NHANES cycles.

## Results

### Study characteristics

The analytic cohort included 1,992 adults (Table 1). Mean age was 66.56 years (SE 0.33) and 41.07% were women; mean BMI and eGFR were 32.41 kg/m² (SE 0.25) and 71.46 mL/min/1.73 m² (SE 0.71), respectively. Across ALI quartiles, participants in the lowest ALI group (Q1) were older than those in Q4 (70.90 vs. 62.86 years), had lower BMI (28.69 vs. 35.76 kg/m²), and had lower eGFR (63.16 vs. 78.20 mL/min/1.73 m²). Inflammatory cell profiles differed markedly: neutrophil counts and NLR were highest in Q1 and lowest in Q4, whereas lymphocyte counts showed the opposite gradient; serum albumin rose modestly from Q1 to Q4. ePWV decreased progressively from Q1 to Q4. Differences were significant for age, BMI, eGFR, albumin, neutrophils, lymphocytes, NLR, ePWV, and follow-up time (all *P* < 0.001).

**Table 1:** Baseline characteristics of the study population according to ALI quartiles

Characteristic	Overall (*n* = 1992)	Q1 (*n* = 498)	Q2 (*n* = 498)	Q3 (*n* = 498)	Q4 (*n* = 498)	*P*-value ^a^
Age, years	66.56 (0.33)	70.90 (0.49)	67.48 (0.54)	65.09 (0.73)	62.86 (0.59)	**< 0.001**
BMI, kg/m²	32.41 (0.25)	28.69 (0.36)	31.91 (0.42)	33.20 (0.40)	35.76 (0.48)	**< 0.001**
eGFR, mL/min/1.73 m²	71.46 (0.71)	63.16 (1.37)	69.27 (1.27)	75.05 (1.40)	78.20 (1.15)	**< 0.001**
HbA1c, %	6.74 (0.04)	6.55 (0.06)	6.67 (0.08)	6.96 (0.11)	6.77 (0.08)	**0.008**
Fasting glucose, mg/dL	136.87 (1.96)	133.89 (3.62)	138.23 (3.93)	143.98 (4.75)	131.03 (2.72)	0.119
Albumin, g/dL	4.12 (0.01)	4.03 (0.02)	4.10 (0.02)	4.16 (0.02)	4.19 (0.02)	**< 0.001**
Neutrophil, ×10⁹/L	4.74 (0.05)	5.89 (0.13)	4.98 (0.08)	4.39 (0.08)	3.74 (0.07)	**< 0.001**
Lymphocyte, ×10⁹/L	1.96 (0.02)	1.40 (0.03)	1.81 (0.03)	2.11 (0.04)	2.50 (0.03)	**< 0.001**
HEI-2015 score	51.94 (0.40)	52.78 (0.90)	51.86 (0.68)	51.05 (0.79)	52.13 (0.99)	0.517
NLR	2.74 (0.05)	4.58 (0.14)	2.80 (0.04)	2.12 (0.02)	1.53 (0.03)	**< 0.001**
ALI	60.40 (0.96)	28.31 (0.44)	47.00 (0.32)	65.32 (0.32)	100.64 (1.26)	**< 0.001**
ePWV, m/s	10.41 (0.05)	10.98 (0.10)	10.56 (0.10)	10.20 (0.11)	9.90 (0.09)	**< 0.001**
Follow-up time, years	7.17 (0.17)	5.82 (0.25)	6.95 (0.28)	7.85 (0.34)	8.03 (0.29)	**< 0.001**
Age group, n (%)						**< 0.001**
<70	1,039 (56.77)	176 (39.96)	241 (52.85)	280 (63.06)	342 (70.82)	
>=70	953 (43.23)	322 (60.04)	257 (47.15)	218 (36.94)	156 (29.18)	
Sex, n (%)						**0.218**
Male	1,179 (58.93)	325 (62.85)	297 (60.08)	293 (58.90)	264 (53.96)	
Female	813 (41.07)	173 (37.15)	201 (39.92)	205 (41.10)	234 (46.04)	
Race/ethnicity, n (%)						**< 0.001**
Non-Hispanic Black	411 (10.56)	64 (5.87)	84 (8.21)	93 (10.03)	170 (18.14)	
Non-Hispanic White	1,049 (75.19)	316 (80.77)	275 (78.35)	257 (74.38)	201 (67.28)	
Mexican American	281 (4.96)	53 (3.89)	81 (5.36)	82 (5.45)	65 (5.07)	
Other Race	251 (9.29)	65 (9.47)	58 (8.08)	66 (10.15)	62 (9.52)	
Marital status, n (%)						**0.146**
Never married	101 (4.29)	17 (2.30)	27 (4.98)	25 (5.92)	32 (3.83)	
Married/Living with a partner	1,159 (63.34)	283 (62.45)	286 (59.54)	298 (64.40)	292 (67.15)	
Divorced/Separated/Widowed	732 (32.38)	198 (35.26)	185 (35.48)	175 (29.67)	174 (29.02)	
Education level, n (%)						**0.305**
Under high school	741 (26.82)	188 (29.11)	180 (24.95)	185 (25.89)	188 (27.51)	
High school	479 (27.38)	120 (26.36)	141 (31.97)	104 (23.91)	114 (27.01)	
More than high school	772 (45.80)	190 (44.54)	177 (43.08)	209 (50.20)	196 (45.48)	
Smoking status, n (%)						**0.498**
Never	793 (38.70)	175 (35.27)	194 (41.10)	205 (36.22)	219 (41.97)	
Former	867 (44.50)	242 (48.29)	219 (43.90)	212 (45.94)	194 (40.00)	
Current	332 (16.81)	81 (16.44)	85 (15.00)	81 (17.84)	85 (18.03)	
Alcohol consumption, n (%)						**0.980**
Never	320 (13.91)	79 (13.71)	76 (15.04)	83 (13.39)	82 (13.41)	
Former	703 (32.43)	191 (32.04)	170 (31.18)	177 (34.28)	165 (32.28)	
Current	969 (53.66)	228 (54.24)	252 (53.78)	238 (52.33)	251 (54.31)	
Hypertension, n (%)						**0.042**
No	321 (16.96)	87 (18.09)	80 (15.29)	89 (21.72)	65 (12.85)	
Yes	1,671 (83.04)	411 (81.91)	418 (84.71)	409 (78.28)	433 (87.15)	
Hyperlipidemia, n (%)						**0.326**
No	165 (6.16)	49 (7.72)	33 (4.98)	44 (6.58)	39 (5.49)	
Yes	1,827 (93.84)	449 (92.28)	465 (95.02)	454 (93.42)	459 (94.51)	
Cancer, n (%)						**0.012**
No	1,583 (77.62)	361 (70.65)	394 (77.65)	408 (80.36)	420 (81.59)	
Yes	409 (22.38)	137 (29.35)	104 (22.35)	90 (19.64)	78 (18.41)	
CVD burden, n (%)						**0.165**
1	1,077 (51.26)	240 (45.94)	270 (48.13)	274 (54.26)	293 (56.70)	
2	493 (25.38)	129 (28.04)	126 (26.18)	124 (25.84)	114 (21.48)	
>=3	422 (23.36)	129 (26.02)	102 (25.68)	100 (19.90)	91 (21.82)	
All-cause mortality, n (%)						**< 0.001**
No	1,081 (58.76)	179 (42.82)	266 (58.60)	292 (60.78)	344 (72.33)	
Yes	911 (41.24)	319 (57.18)	232 (41.40)	206 (39.22)	154 (27.67)	
CVD mortality, n (%)						**0.017**
No	1,624 (83.66)	379 (79.33)	409 (85.22)	403 (81.64)	433 (88.24)	
Yes	368 (16.34)	119 (20.67)	89 (14.78)	95 (18.36)	65 (11.76)	
Premature mortality, n (%)						**0.529**
No	1,679 (84.96)	402 (82.82)	421 (85.56)	425 (84.29)	431 (87.07)	
Yes	313 (15.04)	96 (17.18)	77 (14.44)	73 (15.71)	67 (12.93)	
ePWV quartiles, n (%)						**< 0.001**
Q1	498 (28.38)	85 (19.08)	113 (24.66)	133 (30.33)	167 (39.32)	
Q2	498 (26.77)	100 (22.35)	125 (27.40)	128 (29.11)	145 (28.01)	
Q3	498 (23.45)	129 (24.97)	133 (26.33)	131 (23.98)	105 (18.40)	
Q4	498 (21.41)	184 (33.60)	127 (21.61)	106 (16.58)	81 (14.27)	

Values are presented as survey-weighted mean (SE) for continuous variables and unweighted n (survey-weighted percentage) for categorical variables

^a^: *P*-values were calculated using design-based Wald tests for continuous variables and Rao–Scott adjusted chi-square tests for categorical variables

Race/ethnicity was analyzed as a four-category variable: Non-Hispanic Black, Non-Hispanic White, Mexican American, and Other Race; Other Hispanic and Other Race (including multi-racial) were combined into Other Race

Smoking status was classified as never, former, and current. Alcohol consumption was classified as never, former, and current; moderate, mild, and heavy alcohol use were combined as current alcohol consumption

For premature mortality, formal survival analyses were restricted to participants younger than 75 years at baseline and follow-up was censored at 75 years of age; Table 1 provides descriptive distributions in the analytic cohort

*Abbreviations*: *ALI* advanced lung cancer inflammation index, *ePWV* estimated pulse wave velocity, *CVD* cardiovascular disease, *BMI* body mass index, *eGFR* estimated glomerular filtration rate, *HbA1c* hemoglobin A1c, *HEI*-2015 Healthy Eating Index-2015, *NLR* neutrophil-to-lymphocyte ratio, *SE* standard error

Sociodemographic and clinical patterns also varied by ALI strata. Non-Hispanic White proportions decreased and non-Hispanic Black proportions increased from Q1 through Q4; hypertension was uniformly high with modest variation; a history of cancer was more frequent in Q1 than Q4. In contrast, no significant differences were observed in the distributions of sex, marital status, education, smoking, alcohol consumption, HEI-2015 score, and hyperlipidemia across ALI quartiles (all *P* > 0.05). All-cause and cardiovascular mortality were highest in Q1 and lowest in Q4. Premature mortality was numerically lower in Q4 than in Q1, but the difference across ALI quartiles was not statistically significant.

### Multivariate Cox regression

In the fully adjusted model, higher ALI was consistently associated with lower mortality risk. Each 10-unit increase in ALI was associated with lower risks of all-cause mortality (HR 0.899, 95% CI 0.872–0.928), cardiovascular mortality (HR 0.938, 95% CI 0.885–0.995), and premature mortality (HR 0.889, 95% CI 0.837–0.945) (Table 2). Compared with the lowest ALI quartile, the highest quartile was associated with lower risks of all-cause mortality (HR 0.469, 95% CI 0.372–0.590), cardiovascular mortality (HR 0.578, 95% CI 0.380–0.879), and premature mortality (HR 0.402, 95% CI 0.262–0.616), with significant trends across quartiles.

**Table 2:** Multivariate Cox regression of the associations of ALI and ePWV with all-cause, cardiovascular, and premature mortality

Exposure	Events/*n*	Model 1		Model 2		Model 3	
		HR (95%CI)	*P*-value	HR (95%CI)	*P*-value	HR (95%CI)	*P*-value
All-cause mortality							
ALI (per 10-unit increase)	911/1992	0.849 (0.817, 0.882)	< 0.001	0.877 (0.847, 0.909)	< 0.001	0.899 (0.872, 0.928)	< 0.001
ALI quartiles							
Q1	319/498	Ref.		Ref.		Ref.	
Q2	232/498	0.573 (0.459, 0.714)	< 0.001	0.626 (0.507, 0.774)	< 0.001	0.661 (0.536, 0.814)	< 0.001
Q3	206/498	0.459 (0.365, 0.577)	< 0.001	0.536 (0.427, 0.673)	< 0.001	0.587 (0.471, 0.732)	< 0.001
Q4	154/498	0.317 (0.244, 0.412)	< 0.001	0.400 (0.311, 0.515)	< 0.001	0.469 (0.372, 0.590)	< 0.001
P for trend			< 0.001		< 0.001		< 0.001
ePWV (per 1 m/s increase)	911/1992	1.318 (1.255, 1.384)	< 0.001	1.193 (1.125, 1.266)	< 0.001	1.139 (1.072, 1.211)	< 0.001
ePWV quartiles							
Q1	131/498	Ref.		Ref.		Ref.	
Q2	195/498	1.692 (1.308, 2.190)	< 0.001	1.494 (1.149, 1.944)	0.003	1.333 (1.034, 1.719)	0.027
Q3	256/498	2.709 (2.092, 3.509)	< 0.001	1.841 (1.379, 2.457)	< 0.001	1.464 (1.050, 2.041)	0.025
Q4	329/498	4.127 (3.143, 5.419)	< 0.001	2.501 (1.806, 3.464)	< 0.001	1.928 (1.372, 2.711)	< 0.001
P for trend			< 0.001		< 0.001		< 0.001
CVD mortality							
ALI (per 10-unit increase)	368/1992	0.880 (0.825, 0.939)	< 0.001	0.917 (0.860, 0.977)	0.007	0.938 (0.885, 0.995)	0.032
ALI quartiles							
Q1	119/498	Ref.		Ref.		Ref.	
Q2	89/498	0.566 (0.421, 0.761)	< 0.001	0.627 (0.473, 0.832)	0.001	0.648 (0.481, 0.873)	0.004
Q3	95/498	0.596 (0.429, 0.827)	0.002	0.722 (0.521, 1.000)	0.050	0.761 (0.546, 1.062)	0.108
Q4	65/498	0.372 (0.248, 0.558)	< 0.001	0.497 (0.324, 0.761)	0.001	0.578 (0.380, 0.879)	0.010
P for trend			< 0.001		0.004		0.026
ePWV (per 1 m/s increase)	368/1992	1.385 (1.282, 1.497)	< 0.001	1.269 (1.156, 1.393)	< 0.001	1.216 (1.102, 1.341)	< 0.001
ePWV quartiles							
Q1	45/498	Ref.		Ref.		Ref.	
Q2	84/498	2.110 (1.390, 3.204)	< 0.001	1.780 (1.136, 2.790)	0.012	1.600 (1.035, 2.472)	0.034
Q3	94/498	2.879 (1.838, 4.508)	< 0.001	1.765 (1.027, 3.033)	0.040	1.398 (0.796, 2.457)	0.244
Q4	145/498	5.130 (3.311, 7.949)	< 0.001	2.756 (1.622, 4.684)	< 0.001	2.037 (1.186, 3.498)	0.010
P for trend			< 0.001		< 0.001		0.016
Premature mortality							
ALI (per 10-unit increase)	313/1370	0.857 (0.805, 0.911)	< 0.001	0.856 (0.805, 0.911)	< 0.001	0.889 (0.837, 0.945)	< 0.001
ALI quartiles							
Q1	96/257	Ref.		Ref.		Ref.	
Q2	77/340	0.523 (0.339, 0.808)	0.003	0.530 (0.341, 0.823)	0.005	0.591 (0.396, 0.883)	0.010
Q3	73/357	0.468 (0.307, 0.714)	< 0.001	0.475 (0.311, 0.725)	< 0.001	0.573 (0.388, 0.847)	0.005
Q4	67/416	0.318 (0.204, 0.497)	< 0.001	0.319 (0.202, 0.503)	< 0.001	0.402 (0.262, 0.616)	< 0.001
P for trend			< 0.001		< 0.001		< 0.001
ePWV (per 1 m/s increase)	313/1370	1.134 (1.015, 1.266)	0.026	1.131 (1.003, 1.274)	0.044	1.028 (0.918, 1.151)	0.635
ePWV quartiles							
Q1	122/498	Ref.		Ref.		Ref.	
Q2	121/478	1.376 (0.992, 1.909)	0.056	1.375 (0.984, 1.922)	0.062	1.166 (0.840, 1.618)	0.358
Q3	55/309	1.697 (1.087, 2.650)	0.020	1.678 (1.067, 2.637)	0.025	1.178 (0.707, 1.963)	0.531
Q4	15/85	1.620 (0.700, 3.748)	0.260	1.624 (0.687, 3.839)	0.270	0.951 (0.496, 1.824)	0.881
P for trend			0.009		0.012		0.526

Model 1: unadjusted for any covariates

Model 2: adjusted for age, sex and race/ethnicity

Model 3: adjusted for age, sex, race/ethnicity, marital status, education level, smoking status, alcohol consumption, hypertension, hyperlipidemia, cancer, HEI-2015 and eGFR

Events/n are presented as the number of events divided by the number of participants in each exposure category

For premature mortality, analyses were restricted to participants younger than 75 years at baseline and follow-up was censored at 75 years of age

*Abbreviations*: *Ref* reference, *CVD* cardiovascular disease, *ALI* advanced lung cancer inflammation index, *ePWV* estimated pulse wave velocity, *HR* hazard ratio, *CI* confidence interval, *HEI*-2015 Healthy Eating Index-2015, *eGFR* estimated glomerular filtration rate

Higher ePWV was associated with higher risks of all-cause and cardiovascular mortality. In Model 3, each 1 m/s increase in ePWV was associated with higher risks of all-cause mortality (HR 1.139, 95% CI 1.072–1.211) and cardiovascular mortality (HR 1.216, 95% CI 1.102–1.341), whereas the association with premature mortality was attenuated after full adjustment (HR 1.028, 95% CI 0.918–1.151). Similarly, the highest ePWV quartile was associated with higher all-cause mortality (HR 1.928, 95% CI 1.372–2.711) and cardiovascular mortality (HR 2.037, 95% CI 1.186–3.498), but not premature mortality (HR 0.951, 95% CI 0.496–1.824).

**Fig. 2:**
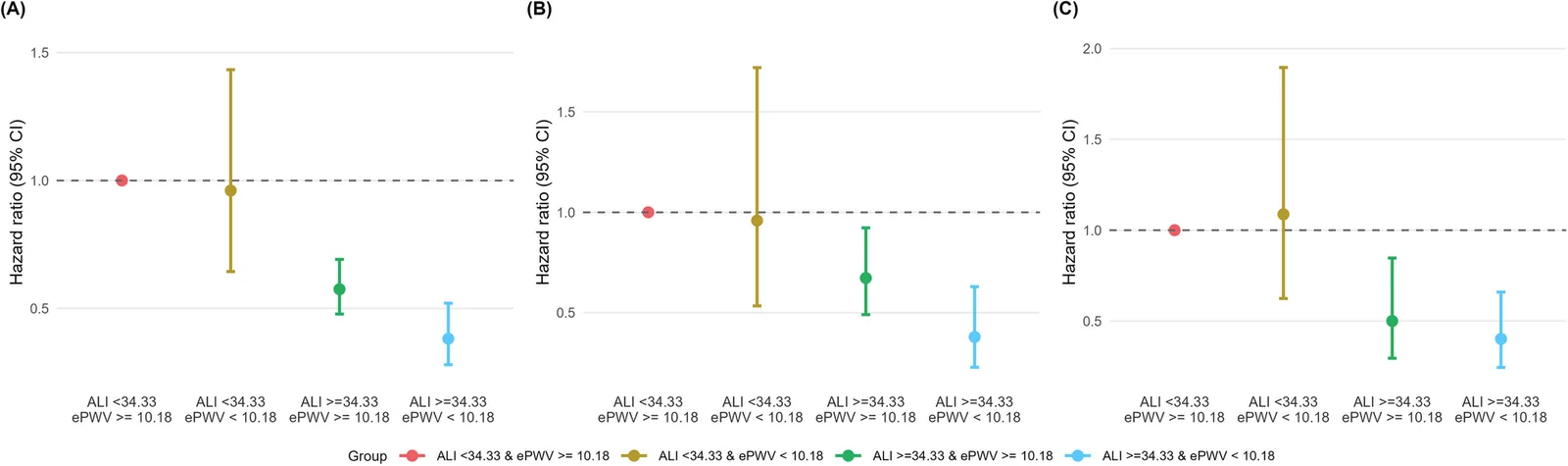
Joint association of ALI and ePWV with all-cause, cardiovascular, and premature mortality. Adjusted for age, sex, race/ethnicity, marital status, education level, smoking status, alcohol consumption, hypertension, hyperlipidemia, cancer, HEI-2015 and eGFR. **A** All-cause mortality; **B** cardiovascular mortality; and **C** premature mortality. Hazard ratios and 95% confidence intervals were estimated using the fully adjusted Cox proportional hazards model, with the low-ALI/high-ePWV group as the reference. Abbreviations: CVD, cardiovascular disease; ALI, advanced lung cancer inflammation index; ePWV, estimated pulse wave velocity; HR, hazard ratio; CI, confidence interval; HEI-2015, Healthy Eating Index-2015; eGFR, estimated glomerular filtration rate

**Fig. 3:**
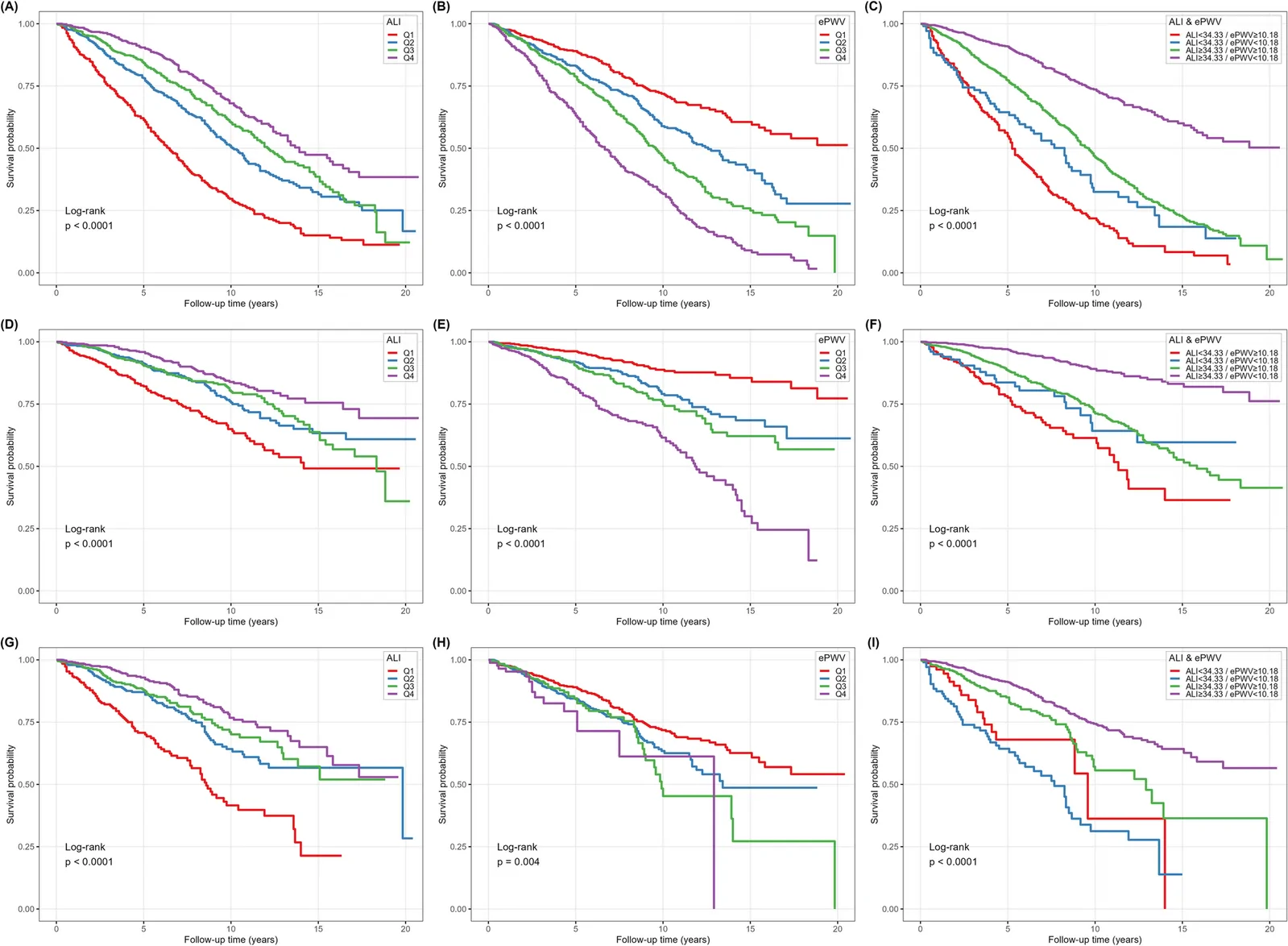
Kaplan–Meier curves of all-cause, cardiovascular, and premature mortality. Panels (**A**-**C**) show the Kaplan–Meier survival curves for all-cause mortality according to ALI quartiles (Q1-Q4), ePWV quartiles (Q1-Q4), and the joint ALI–ePWV categories based on the dichotomized thresholds (ALI 34.33; ePWV 10.18 m/s), respectively. Panels (**D**-**F**) present the same for cardiovascular mortality, while panels (**G**-**I**) show the curves for premature mortality. The *p*-values for all comparisons were calculated using the log-rank test. Abbreviations: CVD, cardiovascular disease; ALI, advanced lung cancer inflammation index; ePWV, estimated pulse wave velocity

Joint ALI–ePWV categories further differentiated mortality risk (Table 3 and Fig. 2). Using the low-ALI/high-ePWV group as the reference, the high-ALI/high-ePWV group had lower risks of all-cause, cardiovascular, and premature mortality. The most favorable profile was high ALI combined with low ePWV, with HRs of 0.382 (95% CI 0.280–0.520) for all-cause mortality, 0.379 (95% CI 0.228–0.630) for cardiovascular mortality, and 0.402 (95% CI 0.245–0.659) for premature mortality. Kaplan–Meier curves showed consistent separation across ALI quartiles, ePWV quartiles, and joint ALI–ePWV categories, with the low-ALI/high-ePWV group showing the least favorable survival pattern and the high-ALI/low-ePWV group showing the most favorable pattern (Fig. 3).

**Table 3:** Joint association of ALI and ePWV with all-cause, cardiovascular, and premature mortality

Exposure	Events/*n*	Model 1		Model 2		Model 3	
		HR (95%CI)	*P*-value	HR (95%CI)	*P*-value	HR (95%CI)	*P*-value
All-cause mortality							
ALI–ePWV joint categories							
ALI < 34.33 & ePWV > = 10.18	188/264	Ref.		Ref.		Ref.	
ALI < 34.33 & ePWV < 10.18	63/103	0.643 (0.431, 0.959)	0.030	0.968 (0.652, 1.439)	0.874	0.961 (0.644, 1.433)	0.845
ALI > = 34.33 & ePWV > = 10.18	478/903	0.493 (0.402, 0.605)	< 0.001	0.525 (0.436, 0.633)	< 0.001	0.575 (0.478, 0.692)	< 0.001
ALI > = 34.33 & ePWV < 10.18	182/722	0.191 (0.143, 0.254)	< 0.001	0.308 (0.228, 0.417)	< 0.001	0.382 (0.280, 0.520)	< 0.001
P for trend			< 0.001		< 0.001		< 0.001
CVD mortality							
ALI–ePWV joint categories							
ALI < 34.33 & ePWV > = 10.18	72/264	Ref.		Ref.		Ref.	
ALI < 34.33 & ePWV < 10.18	23/103	0.612 (0.336, 1.116)	0.109	0.958 (0.521, 1.761)	0.889	0.959 (0.534, 1.721)	0.888
ALI > = 34.33 & ePWV > = 10.18	207/903	0.573 (0.410, 0.801)	0.001	0.617 (0.447, 0.852)	0.003	0.673 (0.490, 0.923)	0.014
ALI > = 34.33 & ePWV < 10.18	66/722	0.185 (0.121, 0.283)	< 0.001	0.315 (0.191, 0.521)	< 0.001	0.379 (0.228, 0.630)	< 0.001
P for trend			< 0.001		< 0.001		< 0.001
Premature mortality							
ALI–ePWV joint categories							
ALI < 34.33 & ePWV > = 10.18	19/77	Ref.		Ref.		Ref.	
ALI < 34.33 & ePWV < 10.18	53/103	1.124 (0.597, 2.113)	0.718	1.141 (0.621, 2.095)	0.671	1.087 (0.624, 1.896)	0.768
ALI > = 34.33 & ePWV > = 10.18	90/473	0.449 (0.243, 0.830)	0.011	0.453 (0.247, 0.828)	0.010	0.500 (0.295, 0.847)	0.010
ALI > = 34.33 & ePWV < 10.18	151/717	0.301 (0.166, 0.547)	< 0.001	0.306 (0.173, 0.542)	< 0.001	0.402 (0.245, 0.659)	< 0.001
P for trend			< 0.001		< 0.001		< 0.001

Model 1: unadjusted for any covariates

Model 2: adjusted for age, sex and race/ethnicity

Model 3: adjusted for age, sex, race/ethnicity, marital status, education level, smoking status, alcohol consumption, hypertension, hyperlipidemia, cancer, HEI-2015 and eGFR

Events/n are presented as the number of events divided by the number of participants in each exposure category

For premature mortality, analyses were restricted to participants younger than 75 years at baseline and follow-up was censored at 75 years of age

*Abbreviations*: *Ref* reference, *CVD* cardiovascular disease, *ALI* advanced lung cancer inflammation index, *ePWV* estimated pulse wave velocity, *HR* hazard ratio, *CI* confidence interval, *HEI*-2015, Healthy Eating Index-2015, *eGFR* estimated glomerular filtration rate

### Nonlinear and threshold relationships between ALI, ePWV, and mortality

**Fig. 4:**
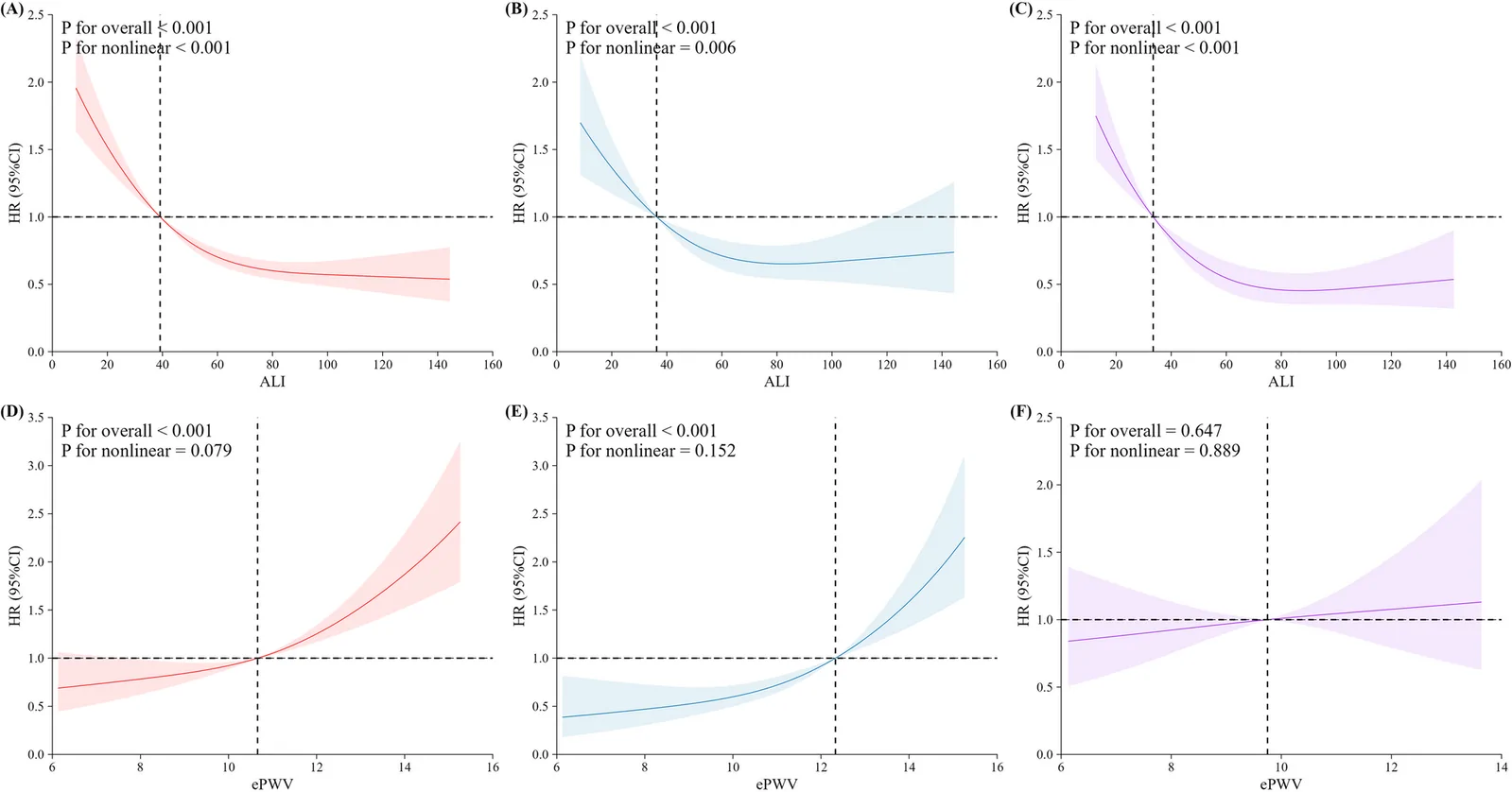
Association between ALI and ePWV with all-cause, cardiovascular and premature mortality using RCS regression. Panels (**A**, **B** and **C**) show HRs for all-cause, CVD and premature mortality by ALI; panels (**D**, **E** and **F**) show the corresponding associations for ePWV. Solid lines represent HRs; shaded areas represent 95% CIs. RCS Cox proportional hazards models were fitted with three knots placed at the 10th, 50th and 90th percentiles of the exposure variable. Adjusted for age, sex, race/ethnicity, marital status, education level, smoking status, alcohol consumption, hypertension, hyperlipidemia, cancer, HEI-2015 and eGFR. Abbreviations: RCS, restricted cubic spline; CVD, cardiovascular disease; ALI, advanced lung cancer inflammation index; ePWV, estimated pulse wave velocity; HR, hazard ratio; CI, confidence interval; HEI-2015, Healthy Eating Index-2015; eGFR, estimated glomerular filtration rate

RCS analyses showed inverse dose–response associations between ALI and all three mortality outcomes (Fig. 4). The risk reduction was steeper at lower ALI values and tended to plateau at higher values, with significant evidence of nonlinearity for all-cause, cardiovascular, and premature mortality. In contrast, ePWV showed generally positive and approximately linear associations with all-cause and cardiovascular mortality, without statistically significant evidence of nonlinearity. No clear association or nonlinear pattern was observed between ePWV and premature mortality after full adjustment.

Threshold analyses were therefore interpreted as exploratory (Table S3). For ALI, the estimated breakpoints were 39.149 for all-cause mortality, 36.295 for cardiovascular mortality, and 33.446 for premature mortality. Below these breakpoints, increasing ALI was more strongly associated with lower mortality risk, whereas the associations were attenuated above the breakpoints. For ePWV, two-piecewise models identified candidate breakpoints around 10–12 m/s; however, likelihood-ratio tests did not provide consistent evidence that these segmented models improved model fit over linear models, particularly for premature mortality. Therefore, the ePWV findings were mainly interpreted as approximately linear associations for all-cause and cardiovascular mortality rather than as threshold effects.

### Discrimination performance of ALI and ePWV

**Fig. 5:**
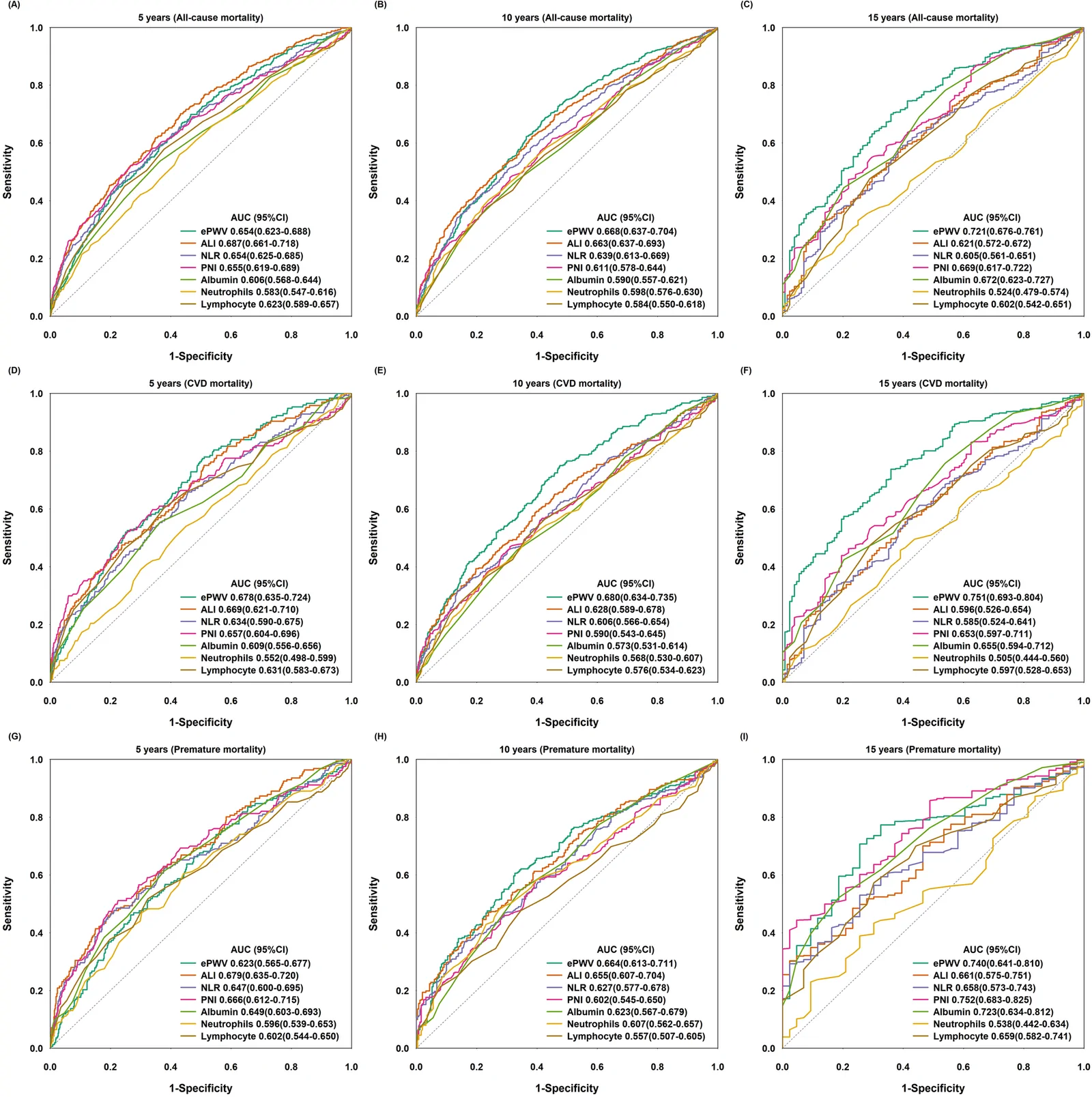
Time-dependent ROC curves for predicting all-cause, cardiovascular, and premature mortality. Panels (**A**-**C**) show the time-dependent ROC curves for all-cause mortality at 5, 10, and 15 years, respectively. Panels (**D**-**F**) present the same for cardiovascular mortality, and panels (**G**-**I**) show the curves for premature death at the same time points. Each curve represents the performance of different models, including ALI quartiles, ePWV quartiles, and other common indices. The AUC values and 95% confidence intervals are shown for each model in the respective subfigure legends.Detailed ROC metrics, including optimal cutoffs, maximum Youden indices, sensitivity, specificity, positive predictive values, negative predictive values, and accuracy, are provided in Table S4. Abbreviations: AUC, area under the curve; ROC, receiver operating characteristic; CVD, cardiovascular disease; ALI, advanced lung cancer inflammation index; ePWV, estimated pulse wave velocity; NLR, neutrophil-to-lymphocyte ratio; PNI, prognostic nutritional index; HR, hazard ratio; CI, confidence interval

Time-dependent ROC analyses indicated modest discrimination across biomarkers and time horizons (Fig. 5). For all-cause mortality, ALI showed the highest 5-year AUC, whereas ePWV showed the highest AUCs at 10 and 15 years. For cardiovascular mortality, ePWV consistently showed the highest AUCs at 5, 10, and 15 years. For premature mortality, the leading marker varied by time horizon: ALI showed the highest 5-year AUC, ePWV showed the highest 10-year AUC, and prognostic nutritional index showed the highest 15-year AUC, with ePWV showing comparable 15-year discrimination. Detailed cutoffs, Youden indices, sensitivity, specificity, positive predictive values, and negative predictive values are provided in Table S4.

**Fig. 6:**
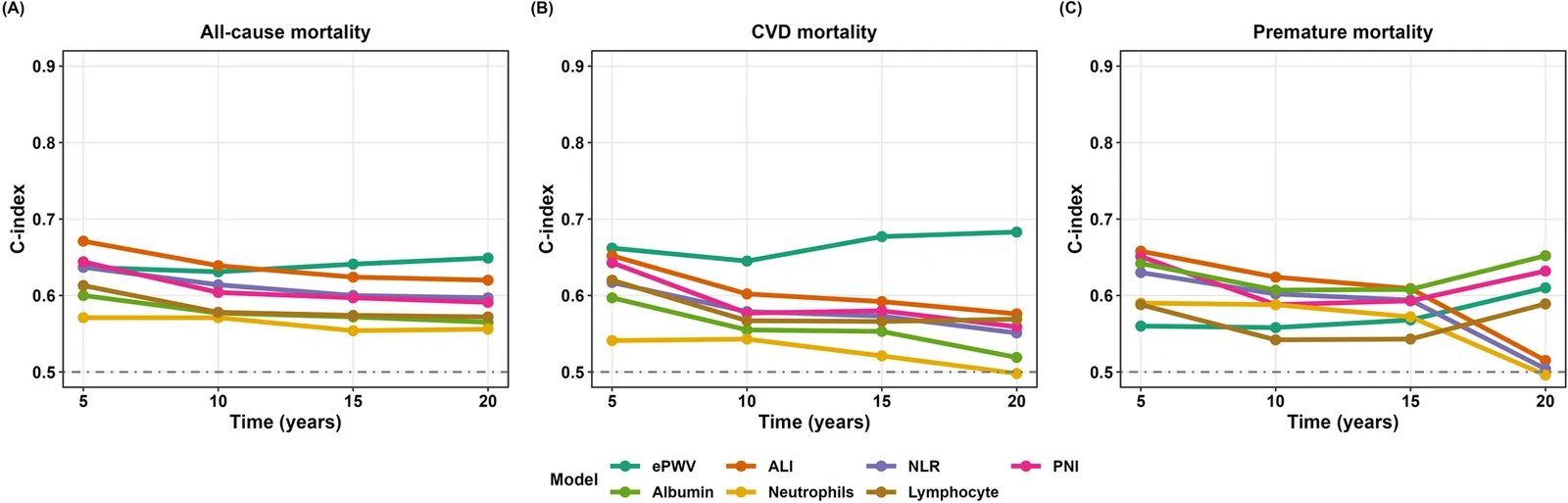
Harrell’s time-dependent C-index curves for all-cause (**A**), cardiovascular (**B**), and premature (**C**) mortality across follow-up (illustrated at 5, 10, 15, and 20 years). Curves compare models using Albumin, ALI, ePWV, lymphocytes, neutrophils, NLR, and PNI. Abbreviations: ALI, advanced lung cancer inflammation index; ePWV, estimated pulse wave velocity; NLR, neutrophil-to-lymphocyte ratio; PNI, prognostic nutritional index

Harrell’s time-dependent C-index curves showed broadly similar patterns (Fig. 6). Overall, ePWV showed stronger discrimination for cardiovascular mortality, whereas ALI retained competitive discrimination for all-cause and premature mortality.

### Subgroup analyses of ALI, ePWV, and their joint categories

**Fig. 7:**
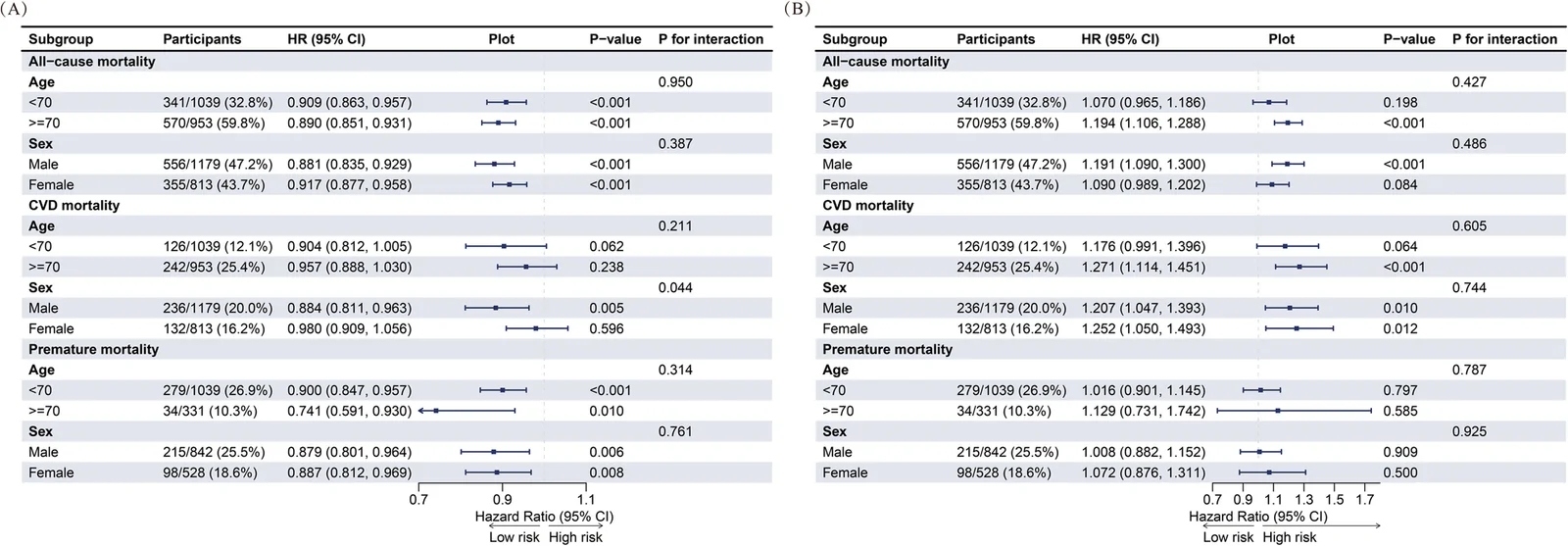
Subgroup analyses of the associations of ALI and ePWV with all-cause, cardiovascular, and premature mortality. **A** Associations of ALI with mortality outcomes stratified by age and sex; **B** associations of ePWV with mortality outcomes stratified by age and sex. Hazard ratios and 95% confidence intervals were estimated using the fully adjusted Cox proportional hazards model. Adjusted for age, sex, race/ethnicity, marital status, education level, smoking status, alcohol consumption, hypertension, hyperlipidemia, cancer, HEI-2015 and eGFR. Abbreviations: CVD, cardiovascular disease; ALI, advanced lung cancer inflammation index; ePWV, estimated pulse wave velocity; HR, hazard ratio; CI, confidence interval; HEI-2015, Healthy Eating Index-2015; eGFR, estimated glomerular filtration rate

Subgroup analyses by age and sex are shown in Fig. 7. The inverse associations between ALI and mortality were generally consistent across strata. A significant sex interaction was observed for cardiovascular mortality, with a stronger inverse association among men (P for interaction = 0.044), whereas no significant age interaction was observed. For ePWV, associations were more apparent for all-cause and cardiovascular mortality than for premature mortality, with no statistically significant interactions by age or sex.

**Fig. 8:**
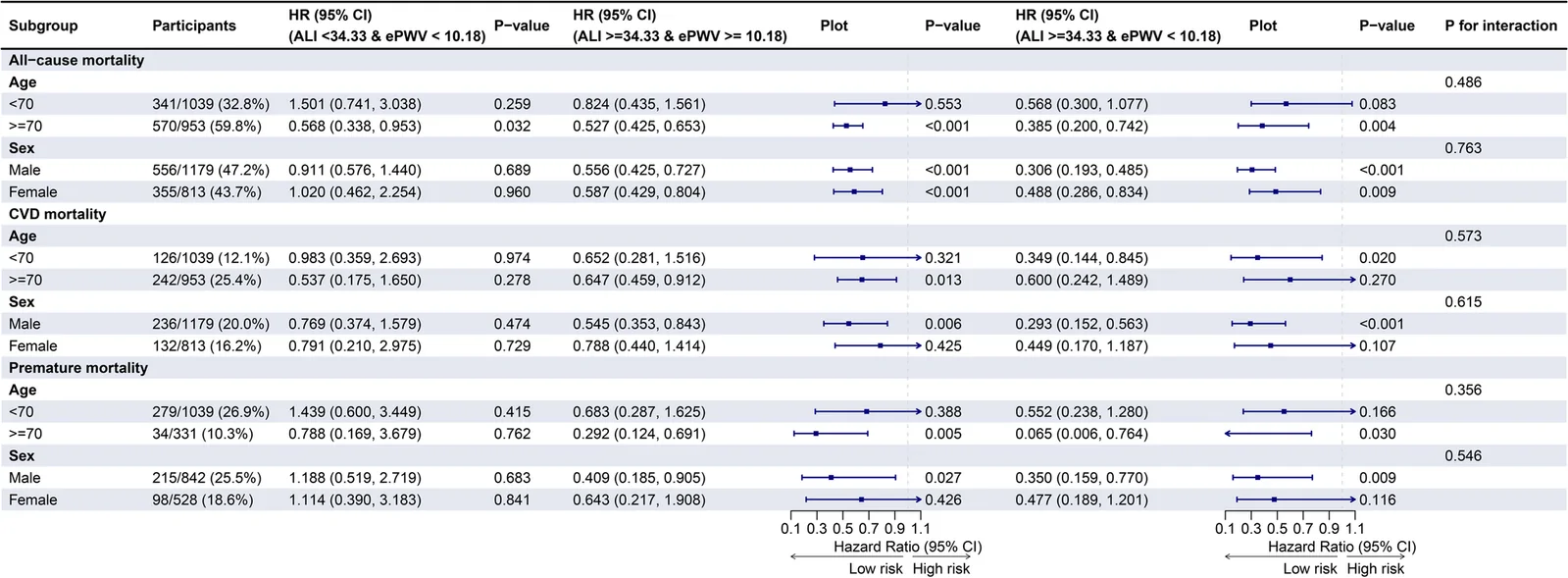
Joint association of ALI and ePWV with all-cause, cardiovascular, and premature mortality across subgroups. Adjusted for age, sex, race/ethnicity, marital status, education level, smoking status, alcohol consumption, hypertension, hyperlipidemia, cancer, HEI-2015 and eGFR. Abbreviations: CVD, cardiovascular disease; ALI, advanced lung cancer inflammation index; ePWV, estimated pulse wave velocity; HR, hazard ratio; CI, confidence interval; HEI-2015, Healthy Eating Index-2015; eGFR, estimated glomerular filtration rate

Joint ALI–ePWV subgroup analyses are shown in Fig. 8. Across age and sex strata, the high-ALI/low-ePWV category generally showed the lowest mortality risk, whereas the low-ALI/high-ePWV category served as the highest-risk reference group. No significant interactions by age or sex were observed for the joint exposure categories.

### Sensitivity analyses

Sensitivity analyses generally supported the robustness of the main findings. Additional adjustment for CVD burden and glycated hemoglobin (HbA1c) produced results similar to the primary fully adjusted models, supporting the stability of the findings after accounting for CVD burden and glycemic control (Table S5). Fasting glucose was not included in this sensitivity model because it was available only in the fasting subsample and had substantial missingness (Table S6).

Including participants with ALI > 150 yielded broadly consistent categorical and joint-exposure results, although the continuous ALI associations were attenuated, supporting the decision to handle these observations as potentially high-leverage values in the primary analysis (Table S7). Excluding deaths within the first two years of follow-up did not materially change the main conclusions (Table S8).

Collinearity diagnostics did not indicate severe multicollinearity, and alternative ePWV adjustment sets yielded generally consistent associations for all-cause and cardiovascular mortality (Table S9-S10). Because Schoenfeld residual tests suggested potential non-proportionality in some models, accelerated failure time models were fitted and supported the main interpretation (Table S11-S12). In Fine–Gray competing-risk models for cardiovascular mortality, ePWV and the joint high-ALI/low-ePWV category remained associated with cardiovascular mortality, whereas the association for ALI alone was attenuated, suggesting that competing non-cardiovascular deaths may partly influence the cause-specific estimates (Table S13).

## Discussion

In this nationally representative cohort of U.S. adults with prediabetes or diabetes and established CVD, higher ALI was associated with lower risks of all-cause, cardiovascular, and premature mortality, whereas higher ePWV was associated with higher all-cause and cardiovascular mortality. When the two markers were considered jointly, the low-ALI/high-ePWV phenotype was associated with the highest mortality risk, whereas the high-ALI/low-ePWV phenotype showed the lowest risk across endpoints. The dose–response analyses suggested an L-shaped association for ALI and a progressively increasing risk pattern for ePWV. Overall, these findings suggest that the joint use of ALI and ePWV may provide a complementary and more integrated approach to characterizing mortality risk in adults with dysglycemia and established CVD.

ALI was originally developed as a composite marker of systemic inflammation and nutritional status in metastatic non-small-cell lung cancer, and lower ALI has been linked to poorer survival in oncologic settings [4, 27]. Beyond oncology, accumulating evidence has associated higher ALI with lower mortality risk in older adults with heart failure, hypertensive populations, cardiovascular–kidney–metabolic phenotypes, and community subgroups such as adults with sarcopenia [5, 7, 8, 28]. Our findings extend this literature to adults with dysglycemia and established CVD, a population in whom inflammation, altered nutritional reserve, adiposity, and metabolic disturbance frequently coexist. The component-level evidence also supports the plausibility of ALI in cardiometabolic risk assessment: serum albumin is related to nutritional reserve and inflammatory burden and has been associated with cardiovascular prognosis, whereas elevated neutrophil-to-lymphocyte ratio reflects immune-inflammatory activation and has been associated with higher all-cause and cardiovascular mortality in CVD populations [29, 30]. Therefore, the inverse association between ALI and mortality in the present study is consistent with the interpretation of ALI as a pragmatic inflammation–nutrition composite, while recognizing that its BMI component also reflects body-size and adiposity-related information.

ePWV has been proposed as a feasible marker of vascular aging when directly measured carotid–femoral pulse wave velocity is unavailable [10, 11]. Previous studies have shown that higher ePWV is associated with all-cause or cardiovascular mortality in the SPRINT trial, general clinical or population-based cohorts, and adults with diabetes from NHANES [12–14, 31, 32]. Our findings are consistent with this literature in showing that higher ePWV was associated with all-cause and cardiovascular mortality. However, the association between ePWV and premature mortality was attenuated after full adjustment, suggesting that vascular-aging-related risk may be more consistently reflected in all-cause and cardiovascular mortality than in premature mortality in this analytic sample. Together with the inverse association observed for ALI, these results highlight the relevance of vascular-aging-related risk as a complementary dimension to inflammation–nutrition status in this high-risk population.

The main contribution of this study is not the evaluation of ALI or ePWV as isolated markers, but the joint assessment of a nutrition–inflammation index and vascular aging in a specific high-risk secondary-prevention population. In addition, the analysis of premature mortality represents a distinct feature of our study, as it extends the outcome assessment beyond conventional all-cause and cardiovascular mortality and provides additional insight into earlier mortality burden in adults with dysglycemia and established CVD. Prior studies have examined inflammation, nutritional status, insulin resistance, vascular aging, frailty, and cardiometabolic mortality as partly overlapping domains, but their combined contribution to mortality risk heterogeneity in adults with dysglycemia and established CVD has been less clearly characterized. Insulin resistance and hyperglycemia contribute to endothelial dysfunction, oxidative stress, lipid dysregulation, low-grade inflammation, and adverse cardiovascular outcomes [3, 17]. Insulin resistance is also closely related to mitochondrial dysfunction, which may further contribute to impaired vascular and metabolic homeostasis [33]. Obesity-related cardiovascular risk appears to be partly mediated through insulin resistance, supporting the biological overlap between adiposity, metabolic dysfunction, and vascular injury [18]. Recent work on cardiovascular–kidney–metabolic syndrome and the interplay between frailty and cardiometabolic disorders further emphasizes that metabolic, vascular, inflammatory, and systemic vulnerability domains often coexist and may amplify adverse outcomes [34, 35]. Evidence that prediabetes increases frailty risk in older adults with hypertension also supports the relevance of early dysglycemia to broader systemic vulnerability [36]. Against this background, the joint ALI–ePWV profile may provide an integrated epidemiological perspective on mortality risk by combining information on inflammation–nutrition status and vascular aging.

The nonlinear analyses provided additional context for these associations. For ALI, the L-shaped curve suggested that mortality risk decreased steeply across the lower range of ALI and then tended to plateau, a pattern broadly consistent with reports in chronic airway disease and cancer-survivor cohorts in which higher ALI was associated with more favorable survival outcomes [37–39]. For ePWV, the RCS curves showed approximately linear positive associations with all-cause and cardiovascular mortality. This pattern is broadly consistent with previous U.S. population-based analyses linking higher ePWV to increased all-cause and cardiovascular mortality [31, 32]. These findings help characterize the shape of the exposure–mortality associations and support evaluating ALI and ePWV both as continuous and grouped exposures, while the data-driven cut-points should be interpreted cautiously.

Subgroup analyses showed generally consistent directions across age and sex strata. A stronger inverse association between ALI and cardiovascular mortality was observed in men, although this finding should be interpreted cautiously given the exploratory nature of subgroup analyses. Sex-related differences in inflammatory tone, body composition, and vascular stiffness biology may partly contribute to this heterogeneity, as prior work has described sex differences in mechanisms of arterial stiffness [40]. Therefore, these subgroup findings should be viewed as hypothesis-generating rather than definitive evidence of sex-specific effects.

From a practical epidemiological perspective, both ALI and ePWV are constructed from routinely collected clinical variables, including anthropometry, albumin, differential counts, age, and blood pressure, minimizing measurement burden and supporting their feasibility for population-based risk characterization. Their joint use leverages distinct yet complementary domains—systemic inflammation/nutritional status and vascular aging—to describe mortality risk heterogeneity. The observed discrimination patterns in the time-dependent ROC and C-index analyses should be interpreted as exploratory evidence of risk discrimination within the study sample rather than proof of clinical prediction utility. While causal inference is beyond the scope of our observational framework, these findings may inform future studies examining whether inflammation–nutrition status and vascular aging provide incremental information beyond conventional clinical factors.

## Strengths and limitations

This study has several strengths. First, NHANES is a nationally representative survey of the civilian, non-institutionalized U.S. population, with standardized interviews, physical examinations, laboratory measurements, and rigorous analytic guidance for complex survey design, supporting population-level inference in adults with dysglycemia and established CVD [41, 42]. Second, vital status was ascertained through probabilistic linkage with the National Death Index, allowing mortality outcomes to be evaluated in a standardized manner [43]. Third, we prespecified covariates based on clinical and epidemiological considerations, incorporated NHANES sampling weights, strata, and primary sampling units, and modeled ALI and ePWV both individually and jointly. We also applied complementary analyses, including survey-weighted Cox models, restricted cubic splines, threshold analyses, time-dependent ROC curves, C-index analyses, subgroup analyses, and multiple sensitivity analyses. These sensitivity analyses included additional adjustment for CVD burden and HbA1c, inclusion of participants with ALI > 150, exclusion of deaths within the first two years of follow-up, collinearity diagnostics, alternative ePWV adjustment models, accelerated failure time models, and Fine–Gray competing-risk models. Together, these analyses strengthened the robustness and interpretability of the findings. Finally, both ALI and ePWV are derived from routinely available clinical measurements, including BMI, serum albumin, differential blood counts, age, and blood pressure, which supports their feasibility for population-level mortality-risk characterization without requiring specialized vascular testing.

Several limitations should also be considered. First, selection bias and generalizability should be interpreted cautiously. NHANES response rates have declined over time, and evidence of nonresponse bias and survey-location variability has been reported in recent cycles; although survey weighting and pooling multiple cycles help mitigate these concerns, residual nonresponse bias may remain [44]. In addition, the primary analyses used a complete-case approach for prespecified covariates. If participants with missing covariates had poorer health status, lower ALI, higher ePWV, and higher mortality risk, the observed associations may have been underestimated; however, if missingness was related to other unmeasured determinants of survival, bias could have occurred in either direction. Second, ALI and ePWV were assessed only once at baseline. Time-varying changes in nutrition–inflammation status, vascular aging, blood pressure, treatment exposure, and metabolic control could not be captured. Within-person variability and measurement error may have introduced exposure misclassification and regression-dilution bias, which would generally attenuate the observed associations toward the null [45, 46]. Third, residual and unmeasured confounding cannot be excluded despite extensive adjustment, which is a common limitation of observational studies [47]. Although medication use was incorporated into the definitions of diabetes, hypertension, and hyperlipidemia when applicable, detailed information on medication classes, treatment intensity, treatment duration, adherence, CVD severity, diabetes duration, and longitudinal glycemic control was not fully available. These unmeasured or incompletely measured factors may have attenuated or exaggerated the observed associations. Disease history and several covariates, including CVD, smoking status, and alcohol consumption, were also based on self-report and may be subject to misclassification. Fourth, interpretation of both exposure markers requires caution. Although ALI has been widely used as a nutrition–inflammation index in oncologic and non-oncologic populations [4–6, 8], it incorporates BMI, serum albumin, and the neutrophil-to-lymphocyte ratio. Therefore, higher ALI should not be interpreted solely as better nutritional or inflammatory status, because it may also reflect body-size- or adiposity-related information. Because ePWV is partly derived from age and blood pressure, adjustment for age and hypertension may raise concerns about collinearity or partial overadjustment. We addressed this issue through collinearity diagnostics and alternative ePWV adjustment models excluding age and hypertension, and the main associations for all-cause and cardiovascular mortality were generally preserved. Nevertheless, fully adjusted ePWV estimates should still be interpreted cautiously because of the structural dependence of ePWV on age and blood pressure. Fifth, the cut-points for ALI and ePWV were selected using maximally selected rank statistics and should be considered exploratory. These thresholds may be sample-specific and susceptible to overfitting, particularly without external validation; therefore, they should not be interpreted as definitive clinical cutoffs. Finally, cardiovascular mortality analyses may be affected by competing non-cardiovascular deaths. Fine–Gray competing-risk models partly addressed this issue, but the attenuation of the ALI association in these models supports cautious interpretation of cause-specific estimates.

## Conclusions

In a nationally representative cohort of U.S. adults with prediabetes/diabetes and cardiovascular disease, higher ALI was independently associated with lower all-cause, cardiovascular, and premature mortality, whereas higher ePWV was associated with higher risk. Joint ALI–ePWV categories delineated clear risk phenotypes (low ALI/high ePWV worst; high ALI/low ePWV best). Because ALI and ePWV are derived from routinely available measurements, their combined assessment may help describe mortality-risk patterns in this population.

## Supplementary Information

Supplementary Material 1.

## Data Availability

Data were utilized from the official NHANES website (https://wwwn.cdc.gov/Nchs/Nhanes/).

## Abbreviations

ADA: American Diabetes Association
AFT: Accelerated failure time
ALI: Advanced lung cancer inflammation index
AUC: Area under the curve
BMI: Body mass index
cfPWV: Carotid–femoral pulse wave velocity
CHF: Congestive heart failure
CHD: Coronary heart disease
C-index: Concordance index
CKD-EPI: Chronic Kidney Disease Epidemiology Collaboration
CVD: Cardiovascular disease
DBP: Diastolic blood pressure
eGFR: Estimated glomerular filtration rate
ePWV: Estimated pulse wave velocity
HbA1c: Hemoglobin A1c
HDL-C: High-density lipoprotein cholesterol
HEI-2015: Healthy Eating Index-2015
ICD-10: International Classification of Diseases, 10th Revision
LDL-C: Low-density lipoprotein cholesterol
LRT: Likelihood ratio test
MBP: Mean blood pressure
MEC: Mobile Examination Center
NCHS: National Center for Health Statistics
NDI: National Death Index
NHANES: National Health and Nutrition Examination Survey
NLR: Neutrophil-to-lymphocyte ratio
NPV: Negative predictive value
OGTT: Oral glucose tolerance test
PH: Proportional hazards
PNI: Prognostic Nutritional Index
PPV: Positive predictive value
PWV: Pulse wave velocity
RCS: Restricted cubic spline
ROC: Receiver operating characteristic
SBP: Systolic blood pressure

## References

[1] Joseph JJ, Deedwania P, Acharya T, Aguilar D, Bhatt DL, Chyun DA, et al. Comprehensive management of cardiovascular risk factors for adults with type 2 diabetes: a scientific statement from the american heart association. Circulation. 2022;145(9):e722–59. 10. 1161/CIR.0000000000001040 PubMed PMID: 35000404. 10.1161/CIR.0000000000001040 35000404

[2] American Diabetes Association Professional Practice Committee. 10. Cardiovascular disease and risk management: standards of care in diabetes-2024. Diabetes Care. 2024;47(Suppl 1):S179–218. 10.2337/dc24-S010 PubMed PMID: 38078592; PubMed Central PMCID: PMC10725811. PMC10725811 38078592

[3] Ormazabal V, Nair S, Elfeky O, Aguayo C, Salomon C, Zuñiga FA. Association between insulin resistance and the development of cardiovascular disease. Cardiovasc Diabetol. 2018;17(1):122. 10.1186/s12933-018-0762-4 . PubMed PMID: 30170598. PMC6119242 30170598

[4] Jafri SH, Shi R, Mills G. Advance lung cancer inflammation index (ALI) at diagnosis is a prognostic marker in patients with metastatic non-small cell lung cancer (NSCLC): a retrospective review. BMC Cancer. 2013;13:158. 10.1186/1471-2407-13-158 . PubMed PMID: 23530866; PubMed Central PMCID: PMC3618002. PMC3618002 23530866

[5] Yuan X, Huang B, Wang R, Tie H, Luo S. The prognostic value of advanced lung cancer inflammation index (ALI) in elderly patients with heart failure. Front Cardiovasc Med. 2022;9:934551. 10.3389/fcvm.2022.934551 . PubMed PMID: 36440019; PubMed Central PMCID: PMC9697177. PMC9697177 36440019

[6] Zhang Y, Pan Y, Tu J, Liao L, Lin S, Chen K, et al. The advanced lung cancer inflammation index predicts long-term outcomes in patients with hypertension: national health and nutrition examination study, 1999–2014. Front Nutr. 2022;9:989914. 10.3389/fnut.2022.989914 . PubMed PMID: 36386913; PubMed Central PMCID: PMC9641016. PMC9641016 36386913

[7] Ma X, Tang X, Chen S, Wu T, Zuo P, Wu F, et al. The Advanced Lung Cancer Inflammation Index and Mortality in Patients with Cardiovascular-Kidney Metabolic Syndrome: Insights from NHANES. BMC Cardiovasc Disord. 2025;25(1):568. 10.1186/s12872-025-05019-6 . PubMed PMID: 40753220; PubMed Central PMCID: PMC12317444. PMC12317444 40753220

[8] Yang Y, Shen S, Luo X, Liu Y, Wang ZX, Li YX, et al. Association of nutritional and inflammatory status with all-cause and cardiovascular mortality in adults with sarcopenia: Insights from NHANES. Maturitas. 2025;196:108233. 10.1016/j.maturitas.2025.108233 . PubMed PMID: 40048843. 40048843

[9] Li X, Zhang Y, He Y, Li KX, Xu RN, Wang H, et al. J-shaped association between serum albumin levels and long-term mortality of cardiovascular disease: experience in national health and nutrition examination survey (2011–2014). Front Cardiovasc Med. 2022;9:1073120. 10.3389/fcvm.2022.1073120 . PubMed PMID: 36523355; PubMed Central PMCID: PMC9745145. PMC9745145 36523355

[10] Greve SV, Laurent S, Olsen MH. Estimated pulse wave velocity calculated from age and mean arterial blood pressure. Pulse (basel Switz). 2017;4(4):175–9. doi:10.1159/000453073 PubMed PMID: 28229052; PubMed Central PMCID: PMC5290427. 10.1159/000453073 PMC5290427 28229052

[11] Boutouyrie P. Estimating is not measuring: the lessons about estimated pulse wave velocity. J Am Heart Assoc. 2022;11(10):e025830. 10.1161. /JAHA.122.025830 PubMed PMID: 35535609; PubMed Central PMCID: PMC9238559. 10.1161/JAHA.122.025830 PMC9238559 35535609

[12] Vlachopoulos C, Terentes-Printzios D, Laurent S, Nilsson PM, Protogerou AD, Aznaouridis K, et al. Association of estimated pulse wave velocity with survival: a secondary analysis of SPRINT. JAMA Netw Open. 2019;2(10):e1912831. 10.1001/jamanetworkopen.2019.12831 . PubMed PMID: 31596491; PubMed Central PMCID: PMC6802234. PMC6802234 31596491

[13] Laugesen E, Olesen KKW, Peters CD, Buus NH, Maeng M, Botker HE, et al. Estimated pulse wave velocity is associated with all-cause mortality during 8.5 years follow-up in patients undergoing elective coronary angiography. J Am Heart Assoc. 2022;11(10):e025173. 10.1161/JAHA.121.025173 . PubMed PMID: 35535599; PubMed Central PMCID: PMC9238554. PMC9238554 35535599

[14] Wu LD, Chu P, Kong CH, Shi Y, Zhu MH, Xia YY, et al. Estimated pulse wave velocity is associated with all-cause mortality and cardiovascular mortality among adults with diabetes. Front Cardiovasc Med. 2023;10:1157163. 10.3389/fcvm.2023.1157163 . PubMed PMID: 37139122; PubMed Central PMCID: PMC10150383. PMC10150383 37139122

[15] Aminuddin A, Lazim MRMLM, Hamid AA, Hui CK, Mohd Yunus MH, Kumar J, et al. The association between inflammation and pulse wave velocity in dyslipidemia: an evidence-based review. Mediators Inflamm. 2020;2020:4732987. 10.1155/2020/4732987 . PubMed PMID: 32908450; PubMed Central PMCID: PMC7450307. PMC7450307 32908450

[16] Aronson D. Cross-linking of glycated collagen in the pathogenesis of arterial and myocardial stiffening of aging and diabetes. J Hypertens. 2003;21(1):3–12. 10.1097/00004872-200301000. -00002 PubMed PMID: 12544424. 12544424

[17] Laakso M, Kuusisto J. Insulin resistance and hyperglycaemia in cardiovascular disease development. Nat Reviews Endocrinol. 2014;10(5):293–302. 10.1038/nrendo.2014.29 . PubMed PMID: 24663222. 24663222

[18] Tian X, Chen S, Wang P, Xu Q, Zhang Y, Luo Y, et al. Insulin resistance mediates obesity-related risk of cardiovascular disease: a prospective cohort study. Cardiovasc Diabetol. 2022;21(1):289. 10.1186/s12933-022-01729-9 . PubMed PMID: 36564775. PMC9789633 36564775

[19] Ray KK, Reuter SB, Dalbeler A, Faurby MD, Letnar G, Mathisen J, et al. Factors Associated with Elevated High-Sensitivity C-Reactive Protein Levels in Individuals with Atherosclerotic Cardiovascular Disease in the US and the UK. Eur J Prev Cardiol. 2025;zwaf609. 10.1093/eurjpc/zwaf609 . PubMed PMID: 41029969. 41029969

[20] Dong G, Gan M, Xu S, Xie Y, Zhou M, Wu L. The neutrophil-lymphocyte ratio as a risk factor for all-cause and cardiovascular mortality among individuals with diabetes: evidence from the NHANES 2003–2016. Cardiovasc Diabetol. 2023;22(1):267. 10.1186/s12933-023-01998-y . PubMed PMID: 37775767; PubMed Central PMCID: PMC10541705. PMC10541705 37775767

[21] Bundy JD, Mills KT, He H, LaVeist TA, Ferdinand KC, Chen J, et al. Social determinants of health and premature death among adults in the USA from 1999 to 2018: a national cohort study. Lancet Public Health. 2023;8(6):e422–31. 10.1016/S2468-2667(. 23)00081-6 PubMed PMID: 37244672; PubMed Central PMCID: PMC10349537. PMC10349537 37244672

[22] Liu Y, Yang S, Wei J. The relationship between visceral adiposity index and estimated pulse wave velocity: insights from NHANES database. Front Nutr. 2025;12:1544084. 10.3389/fnut.2025.1544084 . PubMed PMID: 40568423; PubMed Central PMCID: PMC12189020. PMC12189020 40568423

[23] Krebs-Smith SM, Pannucci TE, Subar AF, Kirkpatrick SI, Lerman JL, Tooze JA, et al. Update of the Healthy Eating Index: HEI-2015. J Acad Nutr Diet. 2018;118(9):1591–602. 10.1016/j.jand.2018.05.021 . PubMed PMID: 30146071; PubMed Central PMCID: PMC6719291. PMC6719291 30146071

[24] Inker LA, Eneanya ND, Coresh J, Tighiouart H, Wang D, Sang Y, et al. New Creatinine- and Cystatin C-Based Equations to Estimate GFR without Race. N Engl J Med. 2021;385(19):1737–49. 10.1056/NEJMoa2102953 . PubMed PMID: 34554658; PubMed Central PMCID: PMC8822996. PMC8822996 34554658

[25] Hosseini Mojahed F, Aalami AH, Pouresmaeil V, Amirabadi A, Qasemi Rad M, Sahebkar A. Clinical Evaluation of the Diagnostic Role of MicroRNA-155 in Breast Cancer. Int J Genomics. 2020;2020:9514831. 10.1155/2020/9514831 . PubMed PMID: 32964011; PubMed Central PMCID: PMC7495225. PMC7495225 32964011

[26] Santulli G, Pascale V, Finelli R, Visco V, Giannotti R, Massari A, et al. We are What We Eat: Impact of Food from Short Supply Chain on Metabolic Syndrome. J Clin Med. 2019;8(12):2061. 10.3390/jcm8122061 . PubMed PMID: 31771147; PubMed Central PMCID: PMC6947359. PMC6947359 31771147

[27] Song M, Zhang Q, Song C, Liu T, Zhang X, Ruan G, et al. The advanced lung cancer inflammation index is the optimal inflammatory biomarker of overall survival in patients with lung cancer. J Cachexia Sarcopenia Muscle. 2022;13(5):2504–14. 10.1002/jcsm.13032 . PubMed PMID: 35833264; PubMed Central PMCID: PMC9530543. PMC9530543 35833264

[28] Tu J, Wu B, Xiu J, Deng J, Lin S, Lu J, et al. Advanced lung cancer inflammation index is associated with long-term cardiovascular death in hypertensive patients: national health and nutrition examination study, 1999–2018. Front Physiol. 2023;14:1074672. 10.3389/fphys.2023.1074672 . PubMed PMID: 37206362; PubMed Central PMCID: PMC10189044. PMC10189044 37206362

[29] Arques S. Serum albumin and cardiovascular disease: state-of-the-art review. Ann Cardiol Angeiol (Paris). 2020;69(4):192–200. 10.1016/j.ancard.2020.07.012 . PubMed PMID: 32797938. 32797938

[30] Li X, Liu M, Wang G. The neutrophil-lymphocyte ratio is associated with all-cause and cardiovascular mortality in cardiovascular patients. Sci Rep. 2024;14(1):26692. 10.1038/s41598-024-76836-6 . PubMed PMID: 39496711; PubMed Central PMCID: PMC11535400. PMC11535400 39496711

[31] Cheng W, Kong F, Pan H, Luan S, Yang S, Chen S. Superior predictive value of estimated pulse wave velocity for all-cause and cardiovascular disease mortality risk in U.S. general adults. BMC Public Health. 2024;24(1):600. 10.1186/s12889-024-18071-2 . PubMed PMID: 38402165; PubMed Central PMCID: PMC10893621. PMC10893621 38402165

[32] Heffernan KS, Jae SY, Loprinzi PD. Association Between Estimated Pulse Wave Velocity and Mortality in U.S. Adults. J Am Coll Cardiol. 2020;75(15):1862–4. 10.1016/j.jacc.2020. 02.035 PubMed PMID: 32299599. 32299599

[33] Mone P, Morgante M, Pansini A, Jankauskas SS, Rizzo M, Lombardi A, et al. Effects of insulin resistance on mitochondrial (dys)function. Atherosclerosis. 2022;341:52–4. 10.1016/j.atherosclerosis.2021.11.026 . PubMed PMID: 34903382; PubMed Central PMCID: PMC8943707. PMC8943707 34903382

[34] Santulli G, Sabatelli G, Wang B, Savino M, Bruno FP, Jankauskas SS, et al. Interplay between frailty and cardiometabolic disorders: from pathophysiology to clinical implications. Cardiovasc Diabetol. 2025;25(1):1. 10.1186/s12933-025-03022-x . PubMed PMID: 41361441; PubMed Central PMCID: PMC12777339. PMC12777339 41361441

[35] Trimarco V, Izzo R, Pacella D, Virginia Manzi M, Trama U, Lembo M, et al. Increased prevalence of cardiovascular-kidney-metabolic syndrome during COVID-19: A propensity score-matched study. Diabetes Res Clin Pract. 2024;218:111926. 10.1016/j.diabres.2024.111926 . PubMed PMID: 39536978. 39536978

[36] Santulli G, Visco V, Varzideh F, Guerra G, Kansakar U, Gasperi M, et al. Prediabetes Increases the Risk of Frailty in Prefrail Older Adults With Hypertension: Beneficial Effects of Metformin. Hypertension. 2024;81(7):1637–43. 10.1161/HYPERTENSIONAHA.124.23087 . PubMed PMID: 38752357; PubMed Central PMCID: PMC11170724. PMC11170724 38752357

[37] Li T, Wang Q, Li Y, Zhang W, Chen M, Deng B, et al. Predictive effects of advanced lung cancer inflammation index and serum vitamin D on mortality in patients with asthma. Nutr J. 2025;24(1):26. 10.1186/s12937-024-01065-6 . PubMed PMID: 39955522; PubMed Central PMCID: PMC11829343. PMC11829343 39955522

[38] Xu Y, Yan Z, Li K, Liu L, Xu L. Association between nutrition-related indicators with the risk of chronic obstructive pulmonary disease and all-cause mortality in the elderly population: evidence from NHANES. Front Nutr. 2024;11:1380791. 10.3389/fnut.2024.1380791 . PubMed PMID: 39081677; PubMed Central PMCID: PMC11286481. PMC11286481 39081677

[39] Yao J, Chen X, Meng F, Cao H, Shu X. Combined influence of nutritional and inflammatory status and depressive symptoms on mortality among US cancer survivors: Findings from the NHANES. Brain Behav Immun. 2024;115:109–17. 10.1016/j.bbi.2023.10.002 . PubMed PMID: 37820973. 37820973

[40] DuPont JJ, Kenney RM, Patel AR, Jaffe IZ. Sex differences in mechanisms of arterial stiffness. Br J Pharmacol. 2019;176(21):4208–25. 10.1111/bph.14624 . PubMed PMID: 30767200; PubMed Central PMCID: PMC6877796. PMC6877796 30767200

[41] Stierman B, Afful J, Carroll MD, Chen TC, Davy O, Fink S, et al. Natl Health Stat Rep. 2021;15810.15620/cdc:106273. PubMed PMID: 39380201; PubMed Central PMCID: PMC11513744. National Health and Nutrition Examination Survey 2017-March 2020 Prepandemic Data Files-Development of Files and Prevalence Estimates for Selected Health Outcomes. 10.15620/cdc:106273 PMC11513744 39380201

[42] Johnson CL, Paulose-Ram R, Ogden CL, Carroll MD, Kruszon-Moran D, Dohrmann SM, et al. National health and nutrition examination survey: analytic guidelines, 1999–2010. Vital Health Stat 2. 2013;(161):1–24. PubMed PMID: 25090154. 25090154

[43] Ingram DD, Lochner KA, Cox CS. Mortality experience of the 1986–2000 National Health Interview Survey Linked Mortality Files participants. Vital Health Stat 2. 2008;(147):1–37. PubMed PMID: 19024797. 19024797

[44] Fakhouri THI, Martin CB, Chen TC, Akinbami LJ, Ogden CL, Paulose-Ram R, et al. An Investigation of Nonresponse Bias and Survey Location Variability in the 2017–2018 National Health and Nutrition Examination Survey. Vital Health Stat 2. 2020;(185):1–36. PubMed PMID: 33541513. 33541513

[45] Clarke R, Shipley M, Lewington S, Youngman L, Collins R, Marmot M, et al. Underestimation of risk associations due to regression dilution in long-term follow-up of prospective studies. Am J Epidemiol. 1999;150(4):341–53. 10.1093/oxfordjournals.aje.a010013 . PubMed PMID: 10453810. 10453810

[46] Frost C, White IR. The effect of measurement error in risk factors that change over time in cohort studies: do simple methods overcorrect for regression dilution? Int J Epidemiol. 2005;34(6):1359–68. 10.1093/ije/dyi148 . PubMed PMID: 16051613. 16051613

[47] Fewell Z, Davey Smith G, Sterne JAC. The impact of residual and unmeasured confounding in epidemiologic studies: a simulation study. Am J Epidemiol. 2007;166(6):646–55. 10.1093/aje/kwm165 . PubMed PMID: 17615092. 17615092

