# Supplementary material for: Associations of a nutrition–inflammation index and vascular aging with mortality in adults with diabetes or prediabetes and cardiovascular disease
Source: BMC Cardiovasc Disord. 2026 Jul 28;26:969. doi: 10.1186/s12872-026-06366-8 (PMC13692866; doi:10.1186/s12872-026-06366-8)
Supplement: Supplementary file 1 [file 12872_2026_6366_MOESM1_ESM.docx]

**Supplementary Material 1**

**Text S1.** Definition of variables involved in this study.

**Sex**

Male; Female.

**Race/ethnicity**

Non-Hispanic White; Non-Hispanic Black; Mexican American; Other race (including other Hispanic and all remaining categories combined per survey cycle).

**Marital status**

Never married; Married/Living with a partner; Divorced/Separated/Widowed.

**Education level**

Under high school; High school; More than high school.

**Smoking status**

Never smoker: reported smoking fewer than 100 cigarettes in the entire lifetime.

Former smoker: smoked at least 100 cigarettes in lifetime but does not smoke now.

Current smoker: smoked at least 100 cigarettes in lifetime and currently smokes “some days” or “every day”.

**Alcohol consumption**

Never: fewer than 12 alcoholic drinks in lifetime.

Former: ≥12 drinks in lifetime but no drinking in the past 12 months (or reported not drinking in the last year).

Current: reported drinking in the past 12 months; intensity classified using sex-specific average intake and binge frequency, informed by NIAAA guidance:

— Mild: women ≤1 drink/day or men ≤2 drinks/day with <2 binge episodes per month.

— Moderate: women 2–3 drinks/day or men 3–4 drinks/day, or 2–4 binge episodes per month.

— Heavy: women ≥4 drinks/day or men ≥5 drinks/day, or ≥5 binge episodes per month.

A standard drink equals 14 g of pure alcohol; binge drinking is defined as ≥4 drinks (women) or ≥5 drinks (men) within ~2 hours.

**Hypertension**

Defined as any of the following: mean systolic blood pressure ≥140 mmHg or mean diastolic blood pressure ≥90 mmHg (averaged across ≥3 seated measurements), use of antihypertensive medications, or self-reported physician diagnosis of hypertension.

**Hyperlipidemia**

Presence of any of the following abnormalities or use of lipid-lowering medication: total cholesterol >=200 mg/dL; LDL-C >=130 mg/dL; triglycerides >=150 mg/dL; HDL-C <40 mg/dL in men or <50 mg/dL in women. Cutoffs reflect NCEP ATP III criteria. Total cholesterol, LDL-C, triglycerides, and HDL-C were identified from NHANES lipid laboratory variables LBXTC, LBDLDL, LBXTR, and LBDHDD, respectively. Current lipid-lowering medication use was identified from the NHANES Blood Pressure and Cholesterol Questionnaire variable BPQ100D and coded as positive when the response was "Yes". Refused, "don't know", and missing responses were treated as missing; participants were classified as having hyperlipidemia if any available laboratory or medication criterion was positive.

**Cancer**

Self-reported physician diagnosis.

**Diet quality (HEI-2015)**

Calculated according to the 13-component HEI-2015 scoring system based on adherence to the Dietary Guidelines for Americans; component definitions and scoring ranges are provided in **Supplementary Table S2**.

**Kidney function (eGFR)**

Estimated glomerular filtration rate was computed using the 2021 CKD-EPI creatinine equation (race-free):

eGFRcr = 142 × min(Scr/k, 1)^a × max(Scr/k, 1)^−1.200 × 0.9938^Age × (1.012 if female), where Scr is serum creatinine (mg/dL), k = 0.7 for females and 0.9 for males, and a = −0.241 for females and −0.302 for males. Creatinine values were IDMS-traceable according to NHANES laboratory standardization.

**Figure S1.** Distributional assessment of ALI before exclusion of extreme values.

Panel A shows the rank-ordered distribution of ALI in the full eligible dataset before excluding participants with extreme ALI values. Panel B shows the same distribution with the x-axis restricted to 0–300 to better visualize the main data range. The red dashed line indicates the predefined extreme-value threshold of ALI = 150 used in the primary analysis. A small number of observations were located far beyond the main distribution, with a maximum ALI value of 2814.32, suggesting potential high-leverage observations. Because ALI is a ratio-based composite index derived from body mass index, serum albumin, and the neutrophil-to-lymphocyte ratio, extremely high values may arise from unusual component combinations rather than the central exposure distribution. Therefore, participants with ALI >150 were excluded from the primary analysis, and sensitivity analyses were performed to assess the robustness of this decision.

**Figure S2.** Determination of the ALI and ePWV cutoff point using maximally selected rank statistics.

Determination of the ALI (A) and ePWV (B) cutoff point using maximally selected rank statistics. Standardized Log-Rank Statistic was utilized in the calculation.

Abbreviations: ALI, advanced lung cancer inflammation index; ePWV, estimated pulse wave velocity.

**Table S1.** Definitions of diabetes, prediabetes, and cardiovascular disease (CVD) in NHANES 1999–2018.

| **Construct** | **Operational definition in this study** | **Sources** |
| --- | --- | --- |
| Diabetes | Any of the following: (a) self-reported physician diagnosis of diabetes or current use of insulin/oral hypoglycemic agents; (b) fasting plasma glucose (FPG) ≥126 mg/dL (7.0 mmol/L); (c) HbA1c ≥6.5% (48 mmol/mol); (d) 2‑hour plasma glucose ≥200 mg/dL during a 75‑g OGTT (applied only in cycles with OGTT). The random plasma glucose criterion (≥200 mg/dL with classic symptoms) is acknowledged in ADA guidance but was not operationalized here due to lack of symptom data in NHANES. | ADA Standards of Care in Diabetes—2024; NHANES laboratory files |
| Prediabetes | Any of the following: (a) self-reported prediabetes/borderline diabetes; (b) FPG 100–125 mg/dL (5.6–6.9 mmol/L); (c) HbA1c 5.7–6.4% (39–46 mmol/mol); (d) 2‑hour plasma glucose 140–199 mg/dL during a 75‑g OGTT (applied only in cycles with OGTT). | ADA Standards of Care in Diabetes—2024; NHANES laboratory files |
| Cardiovascular disease (CVD) | Self-reported physician/health professional diagnosis of any of the following conditions in the Medical Conditions Questionnaire (MCQ series): congestive heart failure (CHF), coronary heart disease (CHD), angina pectoris, myocardial infarction (heart attack), or stroke. | CDC/NCHS NHANES Medical Conditions Questionnaire: MCQ160B (congestive heart failure), MCQ160C (coronary heart disease), MCQ160D (angina/angina pectoris), MCQ160E (heart attack/myocardial infarction), and MCQ160F (stroke). Standard response coding: 1 = Yes, 2 = No, 7 = Refused, 9 = Don't know. |

Definitions follow the American Diabetes Association Standards of Care and the NHANES Medical Conditions Questionnaire. OGTT-based criteria were applied only in survey cycles with available OGTT (primarily 2005–2016).

Abbreviations: ADA, American Diabetes Association; OGTT, oral glucose tolerance test; FPG, fasting plasma glucose; HbA1c, hemoglobin A1c; CHD, coronary heart disease; CHF, congestive heart failure.

Key sources:
1) ADA. 2. Diagnosis and Classification of Diabetes. Diabetes Care. 2024;47(Suppl 1):S20–S44.
2) CDC/NCHS. NHANES Medical Conditions Questionnaire variable list (MCQ160 items).
3) CDC/NCHS. NHANES OGTT laboratory methods documentation (e.g., 2013–2014 cycle).

**Table S2.** Healthy Eating Index-2015 Components & Scoring Standards.

| **Component** | **Maximum points**^1^ | **The standard for maximum score** | **The standard for a minimum score of zero** |
| --- | --- | --- | --- |
| ***Adequacy*** | | | |
| Total Fruits^[2](https://epi.grants.cancer.gov/hei/developing.html#f2)^ | 5 | ≥0.8 cup equiv. per 1,000 kcal | No Fruit |
| Whole Fruits^[3](https://epi.grants.cancer.gov/hei/developing.html#f3)^ | 5 | ≥0.4 cup equiv. per 1,000 kcal | No Whole Fruit |
| Total Vegetables^[4](https://epi.grants.cancer.gov/hei/developing.html#f4)^ | 5 | ≥1.1 cup equiv. per 1,000 kcal | No Vegetables |
| Greens and Beans^[4](https://epi.grants.cancer.gov/hei/developing.html#f4)^ | 5 | ≥0.2 cup equiv. per 1,000 kcal | No Dark Green Vegetables or Legumes |
| Whole Grains | 10 | ≥1.5 oz equiv. per 1,000 kcal | No Whole Grains |
| Dairy^[5](https://epi.grants.cancer.gov/hei/developing.html#f5)^ | 10 | ≥1.3 cup equiv. per 1,000 kcal | No Dairy |
| Total Protein Foods^[6](https://epi.grants.cancer.gov/hei/developing.html#f6)^ | 5 | ≥2.5 oz equiv. per 1,000 kcal | No Protein Foods |
| Seafood and Plant Proteins^[6](https://epi.grants.cancer.gov/hei/developing.html#f6),[7](https://epi.grants.cancer.gov/hei/developing.html#f7)^ | 5 | ≥0.8 oz equiv. per 1,000 kcal | No Seafood or Plant Proteins |
| Fatty Acids^[8](https://epi.grants.cancer.gov/hei/developing.html#f8)^ | 10 | (PUFAs + MUFAs)/SFAs ≥2.5 | (PUFAs + MUFAs)/SFAs ≤1.2 |
| ***Moderation*** | | | |
| Refined Grains | 10 | ≤1.8 oz equiv. per 1,000 kcal | ≥4.3 oz equiv. per 1,000 kcal |
| Sodium | 10 | ≤1.1 gram per 1,000 kcal | ≥2.0 grams per 1,000 kcal |
| Added Sugars | 10 | ≤6.5% of energy | ≥26% of energy |
| Saturated Fats | 10 | ≤8% of energy | ≥16% of energy |

(1) Intakes between the minimum and maximum standards are scored proportionately.

(2) Includes 100% fruit juice.

(3) Includes all forms except juice.

(4) Includes legumes (beans and peas).

(5) Includes all milk products, such as fluid milk, yogurt, cheese, and fortified soy beverages.

(6) Includes legumes (beans and peas).

(7) Includes seafood, nuts, seeds, soy products (other than beverages), and legumes (beans and peas).

(8) Ratio of poly- and monounsaturated fatty acids (PUFAs and MUFAs) to saturated fatty acids (SFAs).

Adequacy components represent the food groups, subgroups, and dietary elements that are encouraged. Higher scores reflect higher intakes for these components because higher intakes are desirable.

Moderation components represent the food groups and dietary elements for which there are recommended limits to consumption. For moderation components, higher scores reflect lower intakes, because lower intakes are more desirable.

**Table S3.** Threshold effect analysis of ALI and ePWV on all-cause, cardiovascular, and premature mortality.

| **Exposure** | **All-cause mortality** | | **CVD mortality** | | **Premature mortality** | |
| --- | --- | --- | --- | --- | --- | --- |
|  | **HR (95%CI)** | **P-value** | **HR (95%CI)** | **P-value** | **HR (95%CI)** | **P-value** |
| **ALI** |  |  |  |  |  |  |
| **Model I** |  |  |  |  |  |  |
| One line effect | 0.989 (0.988, 0.990) | <0.001 | 0.994 (0.991, 0.997) | <0.001 | 0.988 (0.987, 0.990) | <0.001 |
| **Model II** |  |  |  |  |  |  |
| **Turning point(K)** | **39.149** |  | **36.295** |  | **33.446** |  |
| < K effect 1 | 0.967 (0.966, 0.968) | <0.001 | 0.961 (0.957, 0.965) | <0.001 | 0.927 (0.898, 0.957) | <0.001 |
| > K effect 2 | 0.994 (0.993, 0.995) | <0.001 | 0.998 (0.995, 1.001) | 0.287 | 0.994 (0.993, 0.996) | <0.001 |
| LRT test | **<0.001** |  | **<0.001** |  | **<0.001** |  |
| **ePWV** |  |  |  |  |  |  |
| **Model I** |  |  |  |  |  |  |
| One line effect | 1.139 (1.108, 1.172) | <0.001 | 1.216 (1.201, 1.230) | <0.001 | 1.028 (0.986, 1.071) | 0.190 |
| **Model II** |  |  |  |  |  |  |
| **Turning point(K)** | **12.140** |  | **12.328** |  | **12.073** |  |
| < K effect 1 | 1.086 (1.046, 1.127) | <0.001 | 1.117 (1.095, 1.140) | <0.001 | 1.051 (0.992, 1.112) | 0.089 |
| > K effect 2 | 1.264 (1.263, 1.265) | <0.001 | 1.451 (1.437, 1.464) | <0.001 | 0.623 (0.449, 0.864) | 0.005 |
| LRT test | 0.058 |  | 0.051 |  | 0.222 |  |

Adjusted for age, sex, race/ethnicity, marital status, education level, smoking status, alcohol consumption, hypertension, hyperlipidemia, cancer, HEI-2015 and eGFR.

HRs in this threshold-effect table are expressed per 1-unit increase in the original continuous exposure scale, because two-piecewise models were fitted on the original ALI and ePWV scales.

Model I fitted a one-line Cox model. Model II fitted a two-piecewise Cox model using the data-driven turning point (K) selected by the minimum AIC across candidate cut points from the 5th to 95th percentile of each exposure.

The LRT test compares Model II with Model I. For premature mortality, analyses were restricted to participants younger than 75 years at baseline and follow-up was censored at 75 years of age.

Abbreviations: CVD, cardiovascular disease; ALI, advanced lung cancer inflammation index; ePWV, estimated pulse wave velocity; HR, hazard ratio; CI, confidence interval; LRT, log-likelihood ratio test; HEI-2015, Healthy Eating Index-2015; eGFR, estimated glomerular filtration rate.

**Table S4.** Detailed time-dependent ROC metrics for ALI, ePWV, and nutritional-inflammatory biomarkers.

| **Outcome** | **Time point** | **Variable** | **AUC** | **95%CI** | **Time-dependent C-index** | **Bootstrap C-index** | **Best cutoff** | **Youden index (J)** | **Sensitivity** | **Specificity** | **PPV** | **NPV** | **Accuracy** |
| --- | --- | --- | --- | --- | --- | --- | --- | --- | --- | --- | --- | --- | --- |
| All-cause mortality | 5 years | ePWV | 0.654 | (0.623, 0.688) | 0.633 | 0.633 | 10.707 | 0.227 | 0.671 | 0.556 | 0.290 | 0.862 | 0.581 |
|  |  | ALI | 0.687 | (0.661, 0.718) | 0.674 | 0.647 | 54.590 | 0.268 | 0.701 | 0.567 | 0.304 | 0.875 | 0.596 |
|  |  | NLR | 0.654 | (0.625, 0.685) | 0.639 | 0.619 | 2.925 | 0.233 | 0.487 | 0.746 | 0.341 | 0.843 | 0.690 |
|  |  | PNI | 0.655 | (0.619, 0.689) | 0.649 | 0.619 | 7.704 | 0.255 | 0.523 | 0.732 | 0.345 | 0.850 | 0.687 |
|  |  | Albumin | 0.606 | (0.568, 0.644) | 0.607 | 0.589 | 4.074 | 0.173 | 0.537 | 0.636 | 0.285 | 0.836 | 0.615 |
|  |  | Neutrophils | 0.583 | (0.547, 0.616) | 0.573 | 0.568 | 4.593 | 0.131 | 0.559 | 0.571 | 0.260 | 0.828 | 0.569 |
|  |  | Lymphocyte | 0.623 | (0.589, 0.657) | 0.614 | 0.590 | 1.588 | 0.204 | 0.451 | 0.753 | 0.330 | 0.836 | 0.688 |
|  | 10 years | ePWV | 0.668 | (0.637, 0.704) | 0.631 | 0.633 | 10.429 | 0.257 | 0.680 | 0.577 | 0.595 | 0.663 | 0.626 |
|  |  | ALI | 0.663 | (0.637, 0.693) | 0.654 | 0.647 | 54.075 | 0.234 | 0.604 | 0.630 | 0.599 | 0.635 | 0.617 |
|  |  | NLR | 0.639 | (0.613, 0.669) | 0.625 | 0.619 | 2.925 | 0.206 | 0.409 | 0.797 | 0.649 | 0.596 | 0.611 |
|  |  | PNI | 0.611 | (0.578, 0.644) | 0.623 | 0.619 | 7.836 | 0.169 | 0.479 | 0.690 | 0.586 | 0.591 | 0.589 |
|  |  | Albumin | 0.590 | (0.557, 0.621) | 0.591 | 0.589 | 3.993 | 0.137 | 0.348 | 0.789 | 0.602 | 0.570 | 0.578 |
|  |  | Neutrophils | 0.598 | (0.576, 0.630) | 0.573 | 0.568 | 5.108 | 0.163 | 0.404 | 0.759 | 0.606 | 0.582 | 0.589 |
|  |  | Lymphocyte | 0.584 | (0.550, 0.618) | 0.592 | 0.590 | 1.588 | 0.142 | 0.377 | 0.765 | 0.595 | 0.573 | 0.579 |
|  | 15 years | ePWV | 0.721 | (0.676, 0.761) | 0.634 | 0.633 | 10.317 | 0.336 | 0.703 | 0.633 | 0.795 | 0.513 | 0.680 |
|  |  | ALI | 0.621 | (0.572, 0.672) | 0.649 | 0.647 | 39.529 | 0.191 | 0.331 | 0.859 | 0.827 | 0.388 | 0.506 |
|  |  | NLR | 0.605 | (0.561, 0.651) | 0.621 | 0.619 | 2.295 | 0.194 | 0.577 | 0.617 | 0.753 | 0.419 | 0.590 |
|  |  | PNI | 0.669 | (0.617, 0.722) | 0.619 | 0.619 | 8.003 | 0.254 | 0.543 | 0.711 | 0.792 | 0.435 | 0.599 |
|  |  | Albumin | 0.672 | (0.623, 0.727) | 0.588 | 0.589 | 4.398 | 0.243 | 0.782 | 0.461 | 0.746 | 0.511 | 0.676 |
|  |  | Neutrophils | 0.524 | (0.479, 0.574) | 0.569 | 0.568 | 5.211 | 0.081 | 0.331 | 0.750 | 0.728 | 0.357 | 0.469 |
|  |  | Lymphocyte | 0.602 | (0.542, 0.651) | 0.590 | 0.590 | 1.772 | 0.187 | 0.476 | 0.711 | 0.769 | 0.401 | 0.554 |
| CVD mortality | 5 years | ePWV | 0.678 | (0.635, 0.724) | 0.656 | 0.653 | 11.821 | 0.268 | 0.520 | 0.748 | 0.181 | 0.936 | 0.725 |
|  |  | ALI | 0.669 | (0.621, 0.710) | 0.658 | 0.623 | 60.047 | 0.235 | 0.745 | 0.491 | 0.136 | 0.947 | 0.516 |
|  |  | NLR | 0.634 | (0.590, 0.675) | 0.619 | 0.595 | 2.620 | 0.196 | 0.536 | 0.660 | 0.145 | 0.930 | 0.648 |
|  |  | PNI | 0.657 | (0.604, 0.696) | 0.648 | 0.610 | 7.658 | 0.263 | 0.522 | 0.741 | 0.178 | 0.935 | 0.719 |
|  |  | Albumin | 0.609 | (0.556, 0.656) | 0.611 | 0.582 | 4.074 | 0.190 | 0.554 | 0.636 | 0.140 | 0.930 | 0.628 |
|  |  | Neutrophils | 0.552 | (0.498, 0.599) | 0.547 | 0.541 | 4.490 | 0.090 | 0.541 | 0.549 | 0.114 | 0.918 | 0.548 |
|  |  | Lymphocyte | 0.631 | (0.583, 0.673) | 0.619 | 0.591 | 1.772 | 0.226 | 0.604 | 0.622 | 0.146 | 0.936 | 0.620 |
|  | 10 years | ePWV | 0.680 | (0.634, 0.735) | 0.650 | 0.653 | 10.153 | 0.272 | 0.747 | 0.524 | 0.320 | 0.874 | 0.576 |
|  |  | ALI | 0.628 | (0.589, 0.678) | 0.627 | 0.623 | 42.855 | 0.192 | 0.393 | 0.799 | 0.369 | 0.815 | 0.704 |
|  |  | NLR | 0.606 | (0.566, 0.654) | 0.599 | 0.595 | 3.217 | 0.176 | 0.315 | 0.861 | 0.404 | 0.807 | 0.734 |
|  |  | PNI | 0.590 | (0.543, 0.645) | 0.612 | 0.610 | 7.836 | 0.156 | 0.466 | 0.690 | 0.310 | 0.812 | 0.638 |
|  |  | Albumin | 0.573 | (0.531, 0.614) | 0.584 | 0.582 | 4.074 | 0.124 | 0.458 | 0.665 | 0.291 | 0.804 | 0.617 |
|  |  | Neutrophils | 0.568 | (0.530, 0.607) | 0.547 | 0.541 | 5.108 | 0.143 | 0.383 | 0.759 | 0.323 | 0.804 | 0.672 |
|  |  | Lymphocyte | 0.576 | (0.534, 0.623) | 0.593 | 0.591 | 1.864 | 0.140 | 0.564 | 0.575 | 0.285 | 0.815 | 0.573 |
|  | 15 years | ePWV | 0.751 | (0.693, 0.804) | 0.653 | 0.653 | 11.312 | 0.372 | 0.575 | 0.797 | 0.615 | 0.768 | 0.717 |
|  |  | ALI | 0.596 | (0.526, 0.654) | 0.626 | 0.623 | 39.529 | 0.155 | 0.295 | 0.859 | 0.543 | 0.683 | 0.656 |
|  |  | NLR | 0.585 | (0.524, 0.641) | 0.598 | 0.595 | 2.925 | 0.156 | 0.336 | 0.820 | 0.514 | 0.686 | 0.645 |
|  |  | PNI | 0.653 | (0.597, 0.711) | 0.610 | 0.610 | 8.003 | 0.242 | 0.531 | 0.711 | 0.510 | 0.728 | 0.646 |
|  |  | Albumin | 0.655 | (0.594, 0.712) | 0.581 | 0.582 | 4.074 | 0.221 | 0.424 | 0.797 | 0.541 | 0.710 | 0.662 |
|  |  | Neutrophils | 0.505 | (0.444, 0.560) | 0.544 | 0.541 | 4.078 | 0.066 | 0.457 | 0.609 | 0.398 | 0.665 | 0.554 |
|  |  | Lymphocyte | 0.597 | (0.528, 0.653) | 0.592 | 0.591 | 1.772 | 0.185 | 0.474 | 0.711 | 0.481 | 0.705 | 0.625 |
| Premature mortality | 5 years | ePWV | 0.623 | (0.565, 0.677) | 0.553 | 0.550 | 10.065 | 0.188 | 0.462 | 0.726 | 0.213 | 0.894 | 0.689 |
|  |  | ALI | 0.679 | (0.635, 0.720) | 0.665 | 0.645 | 44.302 | 0.265 | 0.477 | 0.788 | 0.265 | 0.904 | 0.744 |
|  |  | NLR | 0.647 | (0.600, 0.695) | 0.638 | 0.624 | 2.936 | 0.264 | 0.478 | 0.786 | 0.264 | 0.904 | 0.743 |
|  |  | PNI | 0.666 | (0.612, 0.715) | 0.664 | 0.630 | 7.658 | 0.282 | 0.499 | 0.783 | 0.270 | 0.907 | 0.744 |
|  |  | Albumin | 0.649 | (0.603, 0.693) | 0.653 | 0.634 | 4.086 | 0.236 | 0.613 | 0.623 | 0.207 | 0.909 | 0.622 |
|  |  | Neutrophils | 0.596 | (0.539, 0.653) | 0.587 | 0.589 | 5.222 | 0.166 | 0.469 | 0.696 | 0.198 | 0.891 | 0.665 |
|  |  | Lymphocyte | 0.602 | (0.544, 0.650) | 0.597 | 0.572 | 1.785 | 0.193 | 0.517 | 0.677 | 0.204 | 0.897 | 0.654 |
|  | 10 years | ePWV | 0.664 | (0.613, 0.711) | 0.550 | 0.550 | 9.184 | 0.274 | 0.621 | 0.653 | 0.480 | 0.770 | 0.642 |
|  |  | ALI | 0.655 | (0.607, 0.704) | 0.647 | 0.645 | 49.693 | 0.228 | 0.471 | 0.757 | 0.499 | 0.735 | 0.659 |
|  |  | NLR | 0.627 | (0.577, 0.678) | 0.624 | 0.624 | 2.957 | 0.210 | 0.369 | 0.841 | 0.544 | 0.721 | 0.680 |
|  |  | PNI | 0.602 | (0.545, 0.650) | 0.631 | 0.630 | 8.064 | 0.187 | 0.581 | 0.606 | 0.431 | 0.737 | 0.597 |
|  |  | Albumin | 0.623 | (0.567, 0.679) | 0.634 | 0.634 | 4.086 | 0.202 | 0.549 | 0.653 | 0.449 | 0.738 | 0.618 |
|  |  | Neutrophils | 0.607 | (0.562, 0.657) | 0.588 | 0.589 | 5.124 | 0.206 | 0.481 | 0.725 | 0.474 | 0.731 | 0.642 |
|  |  | Lymphocyte | 0.557 | (0.507, 0.605) | 0.574 | 0.572 | 1.380 | 0.134 | 0.213 | 0.920 | 0.580 | 0.695 | 0.679 |
|  | 15 years | ePWV | 0.740 | (0.641, 0.810) | 0.550 | 0.550 | 8.789 | 0.451 | 0.707 | 0.744 | 0.702 | 0.749 | 0.727 |
|  |  | ALI | 0.661 | (0.575, 0.751) | 0.646 | 0.645 | 40.566 | 0.280 | 0.303 | 0.977 | 0.917 | 0.622 | 0.667 |
|  |  | NLR | 0.658 | (0.573, 0.743) | 0.623 | 0.624 | 2.407 | 0.269 | 0.525 | 0.744 | 0.636 | 0.648 | 0.643 |
|  |  | PNI | 0.752 | (0.683, 0.825) | 0.631 | 0.630 | 7.851 | 0.389 | 0.435 | 0.953 | 0.888 | 0.665 | 0.715 |
|  |  | Albumin | 0.723 | (0.634, 0.812) | 0.634 | 0.634 | 4.086 | 0.336 | 0.522 | 0.814 | 0.705 | 0.667 | 0.680 |
|  |  | Neutrophils | 0.538 | (0.442, 0.634) | 0.587 | 0.589 | 6.202 | 0.137 | 0.230 | 0.907 | 0.678 | 0.581 | 0.596 |
|  |  | Lymphocyte | 0.659 | (0.582, 0.741) | 0.573 | 0.572 | 2.100 | 0.270 | 0.573 | 0.698 | 0.617 | 0.657 | 0.640 |

AUCs were estimated using cumulative/dynamic time-dependent ROC analysis with inverse probability of censoring weighting.

The time-dependent ROC and C-index analyses are presented as supplementary discrimination analyses; the primary association estimates are based on survey-weighted Cox models.

Figure 5 displays marker-specific AUC values with 95% CIs; this table provides the corresponding cutoffs, Youden index and diagnostic metrics.

The best cutoff was selected by maximizing the Youden index, defined as J = sensitivity + specificity - 1. Higher J indicates a better balance between sensitivity and specificity at the selected time point.

For biomarkers with an inverse risk direction, the marker was multiplied by -1 for ROC estimation to keep AUC values above 0.5; the reported cutoff was converted back to the original marker scale.

For premature mortality, analyses were restricted to participants younger than 75 years at baseline and follow-up was censored at 75 years of age.

Abbreviations: ROC, receiver operating characteristic; AUC, area under the curve; CI, confidence interval; ALI, advanced lung cancer inflammation index; ePWV, estimated pulse wave velocity; NLR, neutrophil-to-lymphocyte ratio; PNI, prognostic nutritional index; PPV, positive predictive value; NPV, negative predictive val.

**Table S5.** Sensitivity analysis with additional adjustment for CVD burden and HbA1c.

| **Exposure** | **Events/n** | **Model 3** | | **Model 3 + CVD burden** | | **Model 3 + CVD burden + HbA1c** | |
| --- | --- | --- | --- | --- | --- | --- | --- |
|  |  | **HR (95%CI)** | **P-value** | **HR (95%CI)** | **P-value** | **HR (95%CI)** | **P-value** |
| **All-cause mortality** |  |  |  |  |  |  |  |
| ALI (per 10-unit increase) | 911/1992 | 0.899 (0.872, 0.928) | **<0.001** | 0.903 (0.876, 0.931) | **<0.001** | 0.900 (0.872, 0.929) | **<0.001** |
| **ALI quartiles** |  |  |  |  |  |  |  |
| Q1 | 319/498 | Ref. |  | Ref. |  | Ref. |  |
| Q2 | 232/498 | 0.661 (0.536, 0.814) | **<0.001** | 0.669 (0.539, 0.830) | **<0.001** | 0.671 (0.540, 0.834) | **<0.001** |
| Q3 | 206/498 | 0.587 (0.471, 0.732) | **<0.001** | 0.593 (0.474, 0.742) | **<0.001** | 0.579 (0.462, 0.726) | **<0.001** |
| Q4 | 154/498 | 0.469 (0.372, 0.590) | **<0.001** | 0.481 (0.382, 0.606) | **<0.001** | 0.469 (0.370, 0.594) | **<0.001** |
| P for trend |  |  | **<0.001** |  | **<0.001** |  | **<0.001** |
| ePWV (per 1 m/s increase) | 911/1992 | 1.139 (1.072, 1.211) | **<0.001** | 1.144 (1.074, 1.218) | **<0.001** | 1.153 (1.082, 1.229) | **<0.001** |
| **ePWV quartiles** |  |  |  |  |  |  |  |
| Q1 | 131/498 | Ref. |  | Ref. |  | Ref. |  |
| Q2 | 195/498 | 1.333 (1.034, 1.719) | **0.027** | 1.343 (1.042, 1.731) | **0.023** | 1.353 (1.054, 1.736) | **0.018** |
| Q3 | 256/498 | 1.464 (1.050, 2.041) | **0.025** | 1.517 (1.087, 2.116) | **0.014** | 1.538 (1.100, 2.150) | **0.012** |
| Q4 | 329/498 | 1.928 (1.372, 2.711) | **<0.001** | 1.985 (1.399, 2.817) | **<0.001** | 2.074 (1.447, 2.973) | **<0.001** |
| P for trend |  |  | **<0.001** |  | **<0.001** |  | **<0.001** |
| **ALI–ePWV joint categories** |  |  |  |  |  |  |  |
| ALI <34.33 & ePWV >= 10.18 | 188/264 | Ref. |  | Ref. |  | Ref. |  |
| ALI <34.33 & ePWV < 10.18 | 63/103 | 0.961 (0.644, 1.433) | 0.845 | 0.932 (0.624, 1.391) | 0.729 | 0.958 (0.640, 1.435) | 0.836 |
| ALI >=34.33 & ePWV >= 10.18 | 478/903 | 0.575 (0.478, 0.692) | **<0.001** | 0.588 (0.487, 0.711) | **<0.001** | 0.591 (0.490, 0.713) | **<0.001** |
| ALI >=34.33 & ePWV < 10.18 | 182/722 | 0.382 (0.280, 0.520) | **<0.001** | 0.387 (0.281, 0.532) | **<0.001** | 0.380 (0.277, 0.520) | **<0.001** |
| P for trend |  |  | **<0.001** |  | **<0.001** |  | **<0.001** |
| **CVD mortality** |  |  |  |  |  |  |  |
| ALI (per 10-unit increase) | 368/1992 | 0.938 (0.885, 0.995) | **0.032** | 0.942 (0.888, 0.998) | **0.043** | 0.936 (0.882, 0.993) | **0.028** |
| **ALI quartiles** |  |  |  |  |  |  |  |
| Q1 | 119/498 | Ref. |  | Ref. |  | Ref. |  |
| Q2 | 89/498 | 0.648 (0.481, 0.873) | **0.004** | 0.653 (0.484, 0.881) | **0.005** | 0.650 (0.481, 0.879) | **0.005** |
| Q3 | 95/498 | 0.761 (0.546, 1.062) | 0.108 | 0.756 (0.544, 1.051) | 0.096 | 0.724 (0.523, 1.001) | 0.050 |
| Q4 | 65/498 | 0.578 (0.380, 0.879) | **0.010** | 0.592 (0.389, 0.901) | **0.014** | 0.563 (0.366, 0.867) | **0.009** |
| P for trend |  |  | **0.026** |  | **0.032** |  | **0.017** |
| ePWV (per 1 m/s increase) | 368/1992 | 1.216 (1.102, 1.341) | **<0.001** | 1.219 (1.101, 1.349) | **<0.001** | 1.239 (1.121, 1.370) | **<0.001** |
| **ePWV quartiles** |  |  |  |  |  |  |  |
| Q1 | 45/498 | Ref. |  | Ref. |  | Ref. |  |
| Q2 | 84/498 | 1.600 (1.035, 2.472) | **0.034** | 1.601 (1.036, 2.473) | **0.034** | 1.614 (1.048, 2.485) | **0.030** |
| Q3 | 94/498 | 1.398 (0.796, 2.457) | 0.244 | 1.421 (0.796, 2.538) | 0.235 | 1.480 (0.820, 2.671) | 0.194 |
| Q4 | 145/498 | 2.037 (1.186, 3.498) | **0.010** | 2.053 (1.162, 3.628) | **0.013** | 2.259 (1.261, 4.045) | **0.006** |
| P for trend |  |  | **0.016** |  | **0.021** |  | **0.008** |
| **ALI–ePWV joint categories** |  |  |  |  |  |  |  |
| ALI <34.33 & ePWV >= 10.18 | 72/264 | Ref. |  | Ref. |  | Ref. |  |
| ALI <34.33 & ePWV < 10.18 | 23/103 | 0.959 (0.534, 1.721) | 0.888 | 0.944 (0.518, 1.722) | 0.851 | 0.991 (0.542, 1.809) | 0.976 |
| ALI >=34.33 & ePWV >= 10.18 | 207/903 | 0.673 (0.490, 0.923) | **0.014** | 0.679 (0.495, 0.933) | **0.017** | 0.680 (0.494, 0.937) | **0.018** |
| ALI >=34.33 & ePWV < 10.18 | 66/722 | 0.379 (0.228, 0.630) | **<0.001** | 0.383 (0.231, 0.636) | **<0.001** | 0.368 (0.224, 0.607) | **<0.001** |
| P for trend |  |  | **<0.001** |  | **<0.001** |  | **<0.001** |
| **Premature mortality** |  |  |  |  |  |  |  |
| ALI (per 10-unit increase) | 313/1370 | 0.889 (0.837, 0.945) | **<0.001** | 0.897 (0.845, 0.952) | **<0.001** | 0.889 (0.836, 0.946) | **<0.001** |
| **ALI quartiles** |  |  |  |  |  |  |  |
| Q1 | 96/257 | Ref. |  | Ref. |  | Ref. |  |
| Q2 | 77/340 | 0.591 (0.396, 0.883) | **0.010** | 0.587 (0.393, 0.875) | **0.009** | 0.580 (0.388, 0.867) | **0.008** |
| Q3 | 73/357 | 0.573 (0.388, 0.847) | **0.005** | 0.591 (0.401, 0.870) | **0.008** | 0.559 (0.380, 0.822) | **0.003** |
| Q4 | 67/416 | 0.402 (0.262, 0.616) | **<0.001** | 0.411 (0.268, 0.629) | **<0.001** | 0.387 (0.250, 0.600) | **<0.001** |
| P for trend |  |  | **<0.001** |  | **<0.001** |  | **<0.001** |
| ePWV (per 1 m/s increase) | 313/1370 | 1.028 (0.918, 1.151) | 0.635 | 1.036 (0.922, 1.164) | 0.551 | 1.041 (0.928, 1.168) | 0.492 |
| **ePWV quartiles** |  |  |  |  |  |  |  |
| Q1 | 122/498 | Ref. |  | Ref. |  | Ref. |  |
| Q2 | 121/478 | 1.166 (0.840, 1.618) | 0.358 | 1.171 (0.840, 1.633) | 0.352 | 1.164 (0.837, 1.620) | 0.367 |
| Q3 | 55/309 | 1.178 (0.707, 1.963) | 0.531 | 1.238 (0.737, 2.077) | 0.420 | 1.222 (0.731, 2.043) | 0.444 |
| Q4 | 15/85 | 0.951 (0.496, 1.824) | 0.881 | 1.139 (0.583, 2.226) | 0.702 | 1.183 (0.591, 2.365) | 0.635 |
| P for trend |  |  | 0.526 |  | 0.333 |  | 0.332 |
| **ALI–ePWV joint categories** |  |  |  |  |  |  |  |
| ALI <34.33 & ePWV >= 10.18 | 19/77 | Ref. |  | Ref. |  | Ref. |  |
| ALI <34.33 & ePWV < 10.18 | 53/103 | 1.087 (0.624, 1.896) | 0.768 | 1.047 (0.595, 1.842) | 0.873 | 1.132 (0.653, 1.963) | 0.659 |
| ALI >=34.33 & ePWV >= 10.18 | 90/473 | 0.500 (0.295, 0.847) | **0.010** | 0.538 (0.310, 0.933) | **0.027** | 0.542 (0.316, 0.929) | **0.026** |
| ALI >=34.33 & ePWV < 10.18 | 151/717 | 0.402 (0.245, 0.659) | **<0.001** | 0.418 (0.251, 0.696) | **<0.001** | 0.415 (0.256, 0.674) | **<0.001** |
| P for trend |  |  | **<0.001** |  | **<0.001** |  | **<0.001** |

Model 3 adjusted for age, sex, race/ethnicity, marital status, education level, smoking status, alcohol consumption, hypertension, hyperlipidemia, cancer, HEI-2015 and eGFR.

The second model additionally adjusted for CVD burden, defined as the number of self-reported CVD conditions and categorized as 1, 2 and ≥3 conditions.

The third model additionally adjusted for both CVD burden and HbA1c. HbA1c was selected for the glycemic-control sensitivity analysis because its missingness was low, whereas fasting glucose was not additionally adjusted because of substantial missingness across pooled NHANES cycles.

Events/n are presented as the number of events divided by the number of participants in each exposure category.

For premature mortality, analyses were restricted to participants younger than 75 years at baseline and follow-up was censored at 75 years of age.

Abbreviations: Ref, reference; CVD, cardiovascular disease; ALI, advanced lung cancer inflammation index; ePWV, estimated pulse wave velocity; HR, hazard ratio; CI, confidence interval; HEI-2015, Healthy Eating Index-2015; eGFR, estimated glomerular filtration rate.

**Table S6.** Missingness summary for key analytic variables.

| **Analytic variable** | **Non-missing before covariate exclusions, n** | **Missing before covariate exclusions, n** | **Missing before covariate exclusions, %** | **Non-missing in primary analytic cohort, n** | **Missing in primary analytic cohort, n** | **Missing in primary analytic cohort, %** |
| --- | --- | --- | --- | --- | --- | --- |
| ALI | 2,359 | 0 | 0.00 | 1,992 | 0 | 0.00 |
| ePWV (per 1 m/s increase) | 2,359 | 0 | 0.00 | 1,992 | 0 | 0.00 |
| Age | 2,359 | 0 | 0.00 | 1,992 | 0 | 0.00 |
| Sex | 2,359 | 0 | 0.00 | 1,992 | 0 | 0.00 |
| Race/ethnicity | 2,359 | 0 | 0.00 | 1,992 | 0 | 0.00 |
| Marital status | 2,350 | 9 | 0.38 | 1,992 | 0 | 0.00 |
| Education level | 2,351 | 8 | 0.34 | 1,992 | 0 | 0.00 |
| Smoking status | 2,358 | 1 | 0.04 | 1,992 | 0 | 0.00 |
| Alcohol consumption | 2,130 | 229 | 9.71 | 1,992 | 0 | 0.00 |
| Hypertension | 2,359 | 0 | 0.00 | 1,992 | 0 | 0.00 |
| Hyperlipidemia | 2,359 | 0 | 0.00 | 1,992 | 0 | 0.00 |
| Cancer | 2,356 | 3 | 0.13 | 1,992 | 0 | 0.00 |
| HEI-2015 | 2,240 | 119 | 5.04 | 1,992 | 0 | 0.00 |
| eGFR | 2,359 | 0 | 0.00 | 1,992 | 0 | 0.00 |
| HbA1c | 2,355 | 4 | 0.17 | 1,988 | 4 | 0.20 |
| Fasting glucose, mg/dL | 1,452 | 907 | 38.45 | 1,230 | 762 | 38.25 |
| Fasting glucose, mmol/L | 1,452 | 907 | 38.45 | 1,230 | 762 | 38.25 |
| CVD burden | 2,359 | 0 | 0.00 | 1,992 | 0 | 0.00 |

Note: This table summarizes missingness and non-missing sample sizes for key exposure and covariate variables before covariate exclusions and in the primary analytic cohort. The dataset before covariate exclusions included participants with complete core exposure data, defined as available ALI and ePWV, after excluding those with missing ALI or ePWV. The primary analytic cohort further excluded participants with missing prespecified Model 3 covariates and extreme ALI values >150. Follow-up time and mortality indicators had no missingness after mortality linkage and are not displayed to avoid redundant rows. HbA1c and CVD burden are displayed because they were used in additional-adjustment sensitivity analyses, but they were not included in the primary Model 3. Fasting glucose was not additionally adjusted because it was available only in the fasting subsample and had substantial missingness across pooled NHANES cycles.

Abbreviations: ALI, advanced lung cancer inflammation index; ePWV, estimated pulse wave velocity; CVD, cardiovascular disease; HEI-2015, Healthy Eating Index-2015; eGFR, estimated glomerular filtration rate.

**Table S7.** Sensitivity analysis including participants with ALI >150 for the associations of ALI, ePWV, and their joint categories with mortality.

| **Exposure** | **Events/n** | **Model 1** | | **Model 2** | | **Model 3** | |
| --- | --- | --- | --- | --- | --- | --- | --- |
|  |  | **HR (95%CI)** | **P-value** | **HR (95%CI)** | **P-value** | **HR (95%CI)** | **P-value** |
| **All-cause mortality** |  |  |  |  |  |  |  |
| ALI (per 10-unit increase) | 935/2052 | 0.906 (0.862, 0.952) | **<0.001** | 0.936 (0.893, 0.982) | **0.006** | 0.956 (0.909, 1.004) | 0.074 |
| **ALI quartiles** |  |  |  |  |  |  |  |
| Q1 | 321/501 | Ref. |  | Ref. |  | Ref. |  |
| Q2 | 236/509 | 0.603 (0.493, 0.738) | **<0.001** | 0.658 (0.541, 0.800) | **<0.001** | 0.664 (0.539, 0.818) | **<0.001** |
| Q3 | 210/519 | 0.456 (0.369, 0.562) | **<0.001** | 0.533 (0.438, 0.648) | **<0.001** | 0.563 (0.454, 0.698) | **<0.001** |
| Q4 | 168/523 | 0.348 (0.273, 0.444) | **<0.001** | 0.455 (0.360, 0.576) | **<0.001** | 0.508 (0.400, 0.645) | **<0.001** |
| P for trend |  |  | **<0.001** |  | **<0.001** |  | **<0.001** |
| ePWV (per 1 m/s increase) | 935/2052 | 1.303 (1.245, 1.364) | **<0.001** | 1.180 (1.117, 1.246) | **<0.001** | 1.144 (1.077, 1.215) | **<0.001** |
| **ePWV quartiles** |  |  |  |  |  |  |  |
| Q1 | 139/530 | Ref. |  | Ref. |  | Ref. |  |
| Q2 | 205/515 | 1.721 (1.333, 2.221) | **<0.001** | 1.496 (1.153, 1.942) | **0.002** | 1.360 (1.046, 1.767) | **0.021** |
| Q3 | 263/512 | 2.626 (2.041, 3.380) | **<0.001** | 1.765 (1.312, 2.375) | **<0.001** | 1.425 (1.022, 1.987) | **0.037** |
| Q4 | 328/495 | 4.095 (3.174, 5.282) | **<0.001** | 2.457 (1.789, 3.375) | **<0.001** | 1.963 (1.399, 2.755) | **<0.001** |
| P for trend |  |  | **<0.001** |  | **<0.001** |  | **<0.001** |
| **ALI–ePWV joint categories** |  |  |  |  |  |  |  |
| ALI <34.33 & ePWV >= 10.18 | 188/264 | Ref. |  | Ref. |  | Ref. |  |
| ALI <34.33 & ePWV < 10.18 | 63/103 | 0.617 (0.421, 0.905) | **0.013** | 0.948 (0.646, 1.392) | 0.787 | 0.958 (0.643, 1.428) | 0.835 |
| ALI >=34.33 & ePWV >= 10.18 | 498/940 | 0.483 (0.400, 0.584) | **<0.001** | 0.521 (0.438, 0.620) | **<0.001** | 0.576 (0.479, 0.692) | **<0.001** |
| ALI >=34.33 & ePWV < 10.18 | 186/745 | 0.194 (0.149, 0.253) | **<0.001** | 0.322 (0.241, 0.429) | **<0.001** | 0.381 (0.280, 0.519) | **<0.001** |
| P for trend |  |  | **<0.001** |  | **<0.001** |  | **<0.001** |
| **CVD mortality** |  |  |  |  |  |  |  |
| ALI (per 10-unit increase) | 378/2052 | 0.918 (0.865, 0.974) | **0.004** | 0.954 (0.901, 1.010) | 0.109 | 0.975 (0.921, 1.033) | 0.395 |
| **ALI quartiles** |  |  |  |  |  |  |  |
| Q1 | 120/501 | Ref. |  | Ref. |  | Ref. |  |
| Q2 | 93/509 | 0.586 (0.441, 0.779) | **<0.001** | 0.648 (0.490, 0.857) | **0.002** | 0.663 (0.489, 0.900) | **0.008** |
| Q3 | 95/519 | 0.582 (0.433, 0.781) | **<0.001** | 0.705 (0.529, 0.939) | **0.017** | 0.753 (0.544, 1.041) | 0.086 |
| Q4 | 70/523 | 0.370 (0.247, 0.554) | **<0.001** | 0.513 (0.335, 0.785) | **0.002** | 0.579 (0.372, 0.900) | **0.015** |
| P for trend |  |  | **<0.001** |  | **0.004** |  | **0.026** |
| ePWV (per 1 m/s increase) | 378/2052 | 1.373 (1.278, 1.475) | **<0.001** | 1.260 (1.156, 1.372) | **<0.001** | 1.219 (1.109, 1.340) | **<0.001** |
| **ePWV quartiles** |  |  |  |  |  |  |  |
| Q1 | 48/530 | Ref. |  | Ref. |  | Ref. |  |
| Q2 | 87/515 | 2.177 (1.427, 3.322) | **<0.001** | 1.834 (1.166, 2.886) | **0.009** | 1.576 (1.008, 2.466) | **0.046** |
| Q3 | 97/512 | 2.996 (1.946, 4.612) | **<0.001** | 1.887 (1.125, 3.164) | **0.016** | 1.435 (0.820, 2.513) | 0.206 |
| Q4 | 146/495 | 5.300 (3.412, 8.232) | **<0.001** | 2.964 (1.744, 5.035) | **<0.001** | 2.189 (1.279, 3.745) | **0.004** |
| P for trend |  |  | **<0.001** |  | **<0.001** |  | **0.005** |
| **ALI–ePWV joint categories** |  |  |  |  |  |  |  |
| ALI <34.33 & ePWV >= 10.18 | 72/264 | Ref. |  | Ref. |  | Ref. |  |
| ALI <34.33 & ePWV < 10.18 | 23/103 | 0.595 (0.334, 1.060) | 0.078 | 0.971 (0.539, 1.750) | 0.923 | 0.963 (0.538, 1.723) | 0.900 |
| ALI >=34.33 & ePWV >= 10.18 | 216/940 | 0.562 (0.407, 0.776) | **<0.001** | 0.615 (0.450, 0.841) | **0.002** | 0.680 (0.496, 0.932) | **0.017** |
| ALI >=34.33 & ePWV < 10.18 | 67/745 | 0.190 (0.129, 0.282) | **<0.001** | 0.343 (0.218, 0.542) | **<0.001** | 0.385 (0.234, 0.634) | **<0.001** |
| P for trend |  |  | **<0.001** |  | **<0.001** |  | **<0.001** |
| **Premature mortality** |  |  |  |  |  |  |  |
| ALI (per 10-unit increase) | 321/1420 | 0.907 (0.859, 0.958) | **<0.001** | 0.908 (0.859, 0.961) | **<0.001** | 0.925 (0.873, 0.979) | **0.007** |
| **ALI quartiles** |  |  |  |  |  |  |  |
| Q1 | 96/259 | Ref. |  | Ref. |  | Ref. |  |
| Q2 | 79/347 | 0.576 (0.381, 0.870) | **0.009** | 0.587 (0.385, 0.893) | **0.013** | 0.573 (0.385, 0.854) | **0.006** |
| Q3 | 77/376 | 0.498 (0.336, 0.740) | **<0.001** | 0.510 (0.344, 0.756) | **<0.001** | 0.543 (0.372, 0.791) | **0.002** |
| Q4 | 69/438 | 0.367 (0.240, 0.560) | **<0.001** | 0.374 (0.241, 0.582) | **<0.001** | 0.408 (0.264, 0.630) | **<0.001** |
| P for trend |  |  | **<0.001** |  | **<0.001** |  | **<0.001** |
| ePWV (per 1 m/s increase) | 321/1420 | 1.142 (1.032, 1.263) | **0.010** | 1.132 (1.016, 1.261) | **0.024** | 1.031 (0.921, 1.153) | 0.598 |
| **ePWV quartiles** |  |  |  |  |  |  |  |
| Q1 | 128/530 | Ref. |  | Ref. |  | Ref. |  |
| Q2 | 121/491 | 1.427 (1.022, 1.992) | **0.037** | 1.425 (1.010, 2.013) | **0.044** | 1.205 (0.868, 1.672) | 0.265 |
| Q3 | 57/313 | 1.863 (1.236, 2.808) | **0.003** | 1.855 (1.214, 2.833) | **0.004** | 1.241 (0.765, 2.013) | 0.382 |
| Q4 | 15/86 | 1.760 (0.880, 3.519) | 0.110 | 1.730 (0.851, 3.516) | 0.130 | 1.063 (0.550, 2.052) | 0.856 |
| P for trend |  |  | **<0.001** |  | **0.001** |  | 0.338 |
| **ALI–ePWV joint categories** |  |  |  |  |  |  |  |
| ALI <34.33 & ePWV >= 10.18 | 19/77 | Ref. |  | Ref. |  | Ref. |  |
| ALI <34.33 & ePWV < 10.18 | 53/103 | 1.044 (0.571, 1.911) | 0.889 | 1.080 (0.607, 1.922) | 0.792 | 1.082 (0.624, 1.874) | 0.780 |
| ALI >=34.33 & ePWV >= 10.18 | 95/500 | 0.441 (0.252, 0.775) | **0.004** | 0.453 (0.263, 0.781) | **0.004** | 0.482 (0.287, 0.809) | **0.006** |
| ALI >=34.33 & ePWV < 10.18 | 154/740 | 0.296 (0.170, 0.515) | **<0.001** | 0.310 (0.185, 0.522) | **<0.001** | 0.392 (0.239, 0.643) | **<0.001** |
| P for trend |  |  | **<0.001** |  | **<0.001** |  | **<0.001** |

Model 1: unadjusted for any covariates.

Model 2: adjusted for age, sex and race/ethnicity.

Model 3: adjusted for age, sex, race/ethnicity, marital status, education level, smoking status, alcohol consumption, hypertension, hyperlipidemia, cancer, HEI-2015 and eGFR.

Events/n are presented as the number of events divided by the number of participants in each exposure category.

For premature mortality, analyses were restricted to participants younger than 75 years at baseline and follow-up was censored at 75 years of age.

Abbreviations: Ref, reference; CVD, cardiovascular disease; ALI, advanced lung cancer inflammation index; ePWV, estimated pulse wave velocity; HR, hazard ratio; CI, confidence interval; HEI-2015, Healthy Eating Index-2015; eGFR, estimated glomerular filtration rate.

**Table S8.** Sensitivity analysis excluding deaths within 2 years for the associations of ALI, ePWV, and their joint categories with mortality.

| **Exposure** | **Events/n** | **Model 1** | | **Model 2** | | **Model 3** | |
| --- | --- | --- | --- | --- | --- | --- | --- |
|  |  | **HR (95%CI)** | **P-value** | **HR (95%CI)** | **P-value** | **HR (95%CI)** | **P-value** |
| **All-cause mortality** |  |  |  |  |  |  |  |
| ALI (per 10-unit increase) | 760/1841 | 0.866 (0.831, 0.903) | **<0.001** | 0.899 (0.864, 0.935) | **<0.001** | 0.918 (0.886, 0.951) | **<0.001** |
| **ALI quartiles** |  |  |  |  |  |  |  |
| Q1 | 243/422 | Ref. |  | Ref. |  | Ref. |  |
| Q2 | 197/463 | 0.607 (0.474, 0.779) | **<0.001** | 0.666 (0.525, 0.846) | **<0.001** | 0.698 (0.552, 0.884) | **0.003** |
| Q3 | 182/474 | 0.484 (0.377, 0.620) | **<0.001** | 0.569 (0.445, 0.728) | **<0.001** | 0.610 (0.481, 0.774) | **<0.001** |
| Q4 | 138/482 | 0.345 (0.260, 0.459) | **<0.001** | 0.448 (0.338, 0.594) | **<0.001** | 0.513 (0.395, 0.666) | **<0.001** |
| P for trend |  |  | **<0.001** |  | **<0.001** |  | **<0.001** |
| ePWV (per 1 m/s increase) | 760/1841 | 1.349 (1.285, 1.417) | **<0.001** | 1.222 (1.151, 1.297) | **<0.001** | 1.175 (1.098, 1.257) | **<0.001** |
| **ePWV quartiles** |  |  |  |  |  |  |  |
| Q1 | 108/475 | Ref. |  | Ref. |  | Ref. |  |
| Q2 | 161/464 | 1.801 (1.398, 2.320) | **<0.001** | 1.593 (1.223, 2.075) | **<0.001** | 1.441 (1.120, 1.854) | **0.005** |
| Q3 | 221/463 | 3.068 (2.397, 3.927) | **<0.001** | 2.126 (1.620, 2.789) | **<0.001** | 1.741 (1.238, 2.448) | **0.001** |
| Q4 | 270/439 | 4.670 (3.588, 6.078) | **<0.001** | 2.898 (2.123, 3.957) | **<0.001** | 2.337 (1.639, 3.333) | **<0.001** |
| P for trend |  |  | **<0.001** |  | **<0.001** |  | **<0.001** |
| **ALI–ePWV joint categories** |  |  |  |  |  |  |  |
| ALI <34.33 & ePWV >= 10.18 | 141/217 | Ref. |  | Ref. |  | Ref. |  |
| ALI <34.33 & ePWV < 10.18 | 44/84 | 0.567 (0.361, 0.890) | **0.014** | 0.862 (0.550, 1.352) | 0.518 | 0.811 (0.498, 1.321) | 0.400 |
| ALI >=34.33 & ePWV >= 10.18 | 416/841 | 0.538 (0.423, 0.684) | **<0.001** | 0.574 (0.459, 0.719) | **<0.001** | 0.616 (0.494, 0.766) | **<0.001** |
| ALI >=34.33 & ePWV < 10.18 | 159/699 | 0.194 (0.144, 0.260) | **<0.001** | 0.320 (0.233, 0.440) | **<0.001** | 0.383 (0.272, 0.538) | **<0.001** |
| P for trend |  |  | **<0.001** |  | **<0.001** |  | **<0.001** |
| **CVD mortality** |  |  |  |  |  |  |  |
| ALI (per 10-unit increase) | 308/1841 | 0.901 (0.845, 0.960) | **0.001** | 0.939 (0.881, 1.001) | 0.052 | 0.954 (0.901, 1.010) | 0.106 |
| **ALI quartiles** |  |  |  |  |  |  |  |
| Q1 | 87/422 | Ref. |  | Ref. |  | Ref. |  |
| Q2 | 78/463 | 0.651 (0.459, 0.924) | **0.016** | 0.722 (0.516, 1.009) | 0.056 | 0.740 (0.524, 1.046) | 0.088 |
| Q3 | 85/474 | 0.642 (0.445, 0.927) | **0.018** | 0.778 (0.538, 1.124) | 0.181 | 0.790 (0.540, 1.157) | 0.227 |
| Q4 | 58/482 | 0.420 (0.274, 0.644) | **<0.001** | 0.565 (0.359, 0.889) | **0.014** | 0.630 (0.407, 0.976) | **0.038** |
| P for trend |  |  | **<0.001** |  | **0.027** |  | 0.062 |
| ePWV (per 1 m/s increase) | 308/1841 | 1.408 (1.307, 1.516) | **<0.001** | 1.308 (1.183, 1.447) | **<0.001** | 1.258 (1.128, 1.402) | **<0.001** |
| **ePWV quartiles** |  |  |  |  |  |  |  |
| Q1 | 38/475 | Ref. |  | Ref. |  | Ref. |  |
| Q2 | 70/464 | 2.323 (1.432, 3.768) | **<0.001** | 1.992 (1.173, 3.383) | **0.011** | 1.811 (1.075, 3.051) | **0.026** |
| Q3 | 80/463 | 3.019 (1.861, 4.897) | **<0.001** | 1.953 (1.069, 3.571) | **0.030** | 1.574 (0.836, 2.963) | 0.160 |
| Q4 | 120/439 | 5.650 (3.649, 8.746) | **<0.001** | 3.229 (1.813, 5.752) | **<0.001** | 2.491 (1.354, 4.585) | **0.003** |
| P for trend |  |  | **<0.001** |  | **<0.001** |  | **0.006** |
| **ALI–ePWV joint categories** |  |  |  |  |  |  |  |
| ALI <34.33 & ePWV >= 10.18 | 52/217 | Ref. |  | Ref. |  | Ref. |  |
| ALI <34.33 & ePWV < 10.18 | 16/84 | 0.586 (0.287, 1.196) | 0.142 | 0.908 (0.436, 1.890) | 0.796 | 0.925 (0.439, 1.947) | 0.837 |
| ALI >=34.33 & ePWV >= 10.18 | 180/841 | 0.653 (0.442, 0.965) | **0.032** | 0.703 (0.479, 1.032) | 0.072 | 0.759 (0.521, 1.105) | 0.149 |
| ALI >=34.33 & ePWV < 10.18 | 60/699 | 0.208 (0.134, 0.324) | **<0.001** | 0.355 (0.201, 0.625) | **<0.001** | 0.418 (0.231, 0.758) | **0.004** |
| P for trend |  |  | **<0.001** |  | **<0.001** |  | **0.002** |
| **Premature mortality** |  |  |  |  |  |  |  |
| ALI (per 10-unit increase) | 245/1298 | 0.884 (0.825, 0.947) | **<0.001** | 0.884 (0.825, 0.947) | **<0.001** | 0.916 (0.857, 0.979) | **0.010** |
| **ALI quartiles** |  |  |  |  |  |  |  |
| Q1 | 66/226 | Ref. |  | Ref. |  | Ref. |  |
| Q2 | 61/321 | 0.590 (0.350, 0.993) | **0.047** | 0.591 (0.348, 1.006) | 0.052 | 0.690 (0.418, 1.140) | 0.148 |
| Q3 | 61/345 | 0.497 (0.305, 0.811) | **0.005** | 0.506 (0.311, 0.825) | **0.006** | 0.620 (0.396, 0.969) | **0.036** |
| Q4 | 57/406 | 0.362 (0.219, 0.601) | **<0.001** | 0.362 (0.217, 0.604) | **<0.001** | 0.460 (0.282, 0.752) | **0.002** |
| P for trend |  |  | **<0.001** |  | **<0.001** |  | **0.002** |
| ePWV (per 1 m/s increase) | 245/1298 | 1.181 (1.048, 1.331) | **0.006** | 1.168 (1.024, 1.333) | **0.021** | 1.061 (0.939, 1.199) | 0.339 |
| **ePWV quartiles** |  |  |  |  |  |  |  |
| Q1 | 99/475 | Ref. |  | Ref. |  | Ref. |  |
| Q2 | 94/450 | 1.497 (1.075, 2.086) | **0.017** | 1.478 (1.047, 2.086) | **0.026** | 1.251 (0.901, 1.738) | 0.181 |
| Q3 | 41/293 | 1.988 (1.237, 3.193) | **0.005** | 1.926 (1.193, 3.109) | **0.007** | 1.333 (0.767, 2.317) | 0.308 |
| Q4 | 11/80 | 2.247 (0.941, 5.370) | 0.068 | 2.141 (0.850, 5.393) | 0.106 | 1.216 (0.594, 2.490) | 0.592 |
| P for trend |  |  | **<0.001** |  | **0.002** |  | 0.189 |
| **ALI–ePWV joint categories** |  |  |  |  |  |  |  |
| ALI <34.33 & ePWV >= 10.18 | 12/69 | Ref. |  | Ref. |  | Ref. |  |
| ALI <34.33 & ePWV < 10.18 | 34/84 | 0.970 (0.416, 2.263) | 0.944 | 1.022 (0.453, 2.304) | 0.959 | 0.940 (0.431, 2.047) | 0.876 |
| ALI >=34.33 & ePWV >= 10.18 | 68/449 | 0.484 (0.210, 1.115) | 0.088 | 0.491 (0.218, 1.109) | 0.087 | 0.543 (0.249, 1.185) | 0.125 |
| ALI >=34.33 & ePWV < 10.18 | 131/696 | 0.308 (0.138, 0.690) | **0.004** | 0.325 (0.151, 0.699) | **0.004** | 0.430 (0.206, 0.896) | **0.024** |
| P for trend |  |  | **<0.001** |  | **<0.001** |  | **<0.001** |

Model 1: unadjusted for any covariates.

Model 2: adjusted for age, sex and race/ethnicity.

Model 3: adjusted for age, sex, race/ethnicity, marital status, education level, smoking status, alcohol consumption, hypertension, hyperlipidemia, cancer, HEI-2015 and eGFR.

Events/n are presented as the number of events divided by the number of participants in each exposure category.

For premature mortality, analyses were restricted to participants younger than 75 years at baseline and follow-up was censored at 75 years of age.

Abbreviations: Ref, reference; CVD, cardiovascular disease; ALI, advanced lung cancer inflammation index; ePWV, estimated pulse wave velocity; HR, hazard ratio; CI, confidence interval; HEI-2015, Healthy Eating Index-2015; eGFR, estimated glomerular filtration rate.

**Table S9.** Detailed collinearity diagnostics for the fully adjusted Cox models.

| **Outcome** | **Exposure model** | **Variable role** | **Variable** | **VIF/GVIF** | **Df** | **GVIF adjusted** |
| --- | --- | --- | --- | --- | --- | --- |
| All-cause mortality | ALI (per 10-unit increase) | Core exposure | ALI (per 10-unit increase) | 1.105 | 1 | 1.051 |
|  |  | Covariate | Age | 1.336 | 1 | 1.156 |
|  |  | Covariate | Sex | 1.307 | 1 | 1.143 |
|  |  | Covariate | Race/ethnicity | 1.369 | 3 | 1.054 |
|  |  | Covariate | Marital status | 1.254 | 2 | 1.058 |
|  |  | Covariate | Education level | 1.176 | 2 | 1.041 |
|  |  | Covariate | Smoking status | 1.368 | 2 | 1.082 |
|  |  | Covariate | Alcohol consumption | 1.284 | 2 | 1.064 |
|  |  | Covariate | Hypertension | 1.073 | 1 | 1.036 |
|  |  | Covariate | Hyperlipidemia | 1.043 | 1 | 1.021 |
|  |  | Covariate | Cancer | 1.070 | 1 | 1.034 |
|  |  | Covariate | HEI-2015 | 1.101 | 1 | 1.049 |
|  |  | Covariate | eGFR | 1.203 | 1 | 1.097 |
|  | ePWV | Core exposure | ePWV (per 1 m/s increase) | 2.002 | 1 | 1.415 |
|  |  | Covariate | Age | 2.070 | 1 | 1.439 |
|  |  | Covariate | Sex | 1.326 | 1 | 1.151 |
|  |  | Covariate | Race/ethnicity | 1.338 | 3 | 1.050 |
|  |  | Covariate | Marital status | 1.288 | 2 | 1.065 |
|  |  | Covariate | Education level | 1.178 | 2 | 1.042 |
|  |  | Covariate | Smoking status | 1.402 | 2 | 1.088 |
|  |  | Covariate | Alcohol consumption | 1.284 | 2 | 1.065 |
|  |  | Covariate | Hypertension | 1.122 | 1 | 1.059 |
|  |  | Covariate | Hyperlipidemia | 1.052 | 1 | 1.025 |
|  |  | Covariate | Cancer | 1.068 | 1 | 1.034 |
|  |  | Covariate | HEI-2015 | 1.102 | 1 | 1.050 |
|  |  | Covariate | eGFR | 1.183 | 1 | 1.088 |
|  | ALI–ePWV joint categories | Core exposure | ALI–ePWV joint categories | 2.006 | 3 | 1.123 |
|  |  | Covariate | Age | 2.049 | 1 | 1.432 |
|  |  | Covariate | Sex | 1.315 | 1 | 1.147 |
|  |  | Covariate | Race/ethnicity | 1.363 | 3 | 1.053 |
|  |  | Covariate | Marital status | 1.277 | 2 | 1.063 |
|  |  | Covariate | Education level | 1.196 | 2 | 1.046 |
|  |  | Covariate | Smoking status | 1.395 | 2 | 1.087 |
|  |  | Covariate | Alcohol consumption | 1.292 | 2 | 1.066 |
|  |  | Covariate | Hypertension | 1.097 | 1 | 1.047 |
|  |  | Covariate | Hyperlipidemia | 1.046 | 1 | 1.023 |
|  |  | Covariate | Cancer | 1.078 | 1 | 1.038 |
|  |  | Covariate | HEI-2015 | 1.108 | 1 | 1.052 |
|  |  | Covariate | eGFR | 1.184 | 1 | 1.088 |
| CVD mortality | ALI (per 10-unit increase) | Core exposure | ALI (per 10-unit increase) | 1.110 | 1 | 1.054 |
|  |  | Covariate | Age | 1.323 | 1 | 1.150 |
|  |  | Covariate | Sex | 1.311 | 1 | 1.145 |
|  |  | Covariate | Race/ethnicity | 1.336 | 3 | 1.050 |
|  |  | Covariate | Marital status | 1.247 | 2 | 1.057 |
|  |  | Covariate | Education level | 1.178 | 2 | 1.042 |
|  |  | Covariate | Smoking status | 1.358 | 2 | 1.080 |
|  |  | Covariate | Alcohol consumption | 1.292 | 2 | 1.066 |
|  |  | Covariate | Hypertension | 1.070 | 1 | 1.035 |
|  |  | Covariate | Hyperlipidemia | 1.031 | 1 | 1.016 |
|  |  | Covariate | Cancer | 1.070 | 1 | 1.034 |
|  |  | Covariate | HEI-2015 | 1.102 | 1 | 1.050 |
|  |  | Covariate | eGFR | 1.198 | 1 | 1.094 |
|  | ePWV | Core exposure | ePWV (per 1 m/s increase) | 1.967 | 1 | 1.402 |
|  |  | Covariate | Age | 2.032 | 1 | 1.426 |
|  |  | Covariate | Sex | 1.338 | 1 | 1.157 |
|  |  | Covariate | Race/ethnicity | 1.316 | 3 | 1.047 |
|  |  | Covariate | Marital status | 1.289 | 2 | 1.066 |
|  |  | Covariate | Education level | 1.180 | 2 | 1.042 |
|  |  | Covariate | Smoking status | 1.381 | 2 | 1.084 |
|  |  | Covariate | Alcohol consumption | 1.285 | 2 | 1.065 |
|  |  | Covariate | Hypertension | 1.120 | 1 | 1.058 |
|  |  | Covariate | Hyperlipidemia | 1.046 | 1 | 1.023 |
|  |  | Covariate | Cancer | 1.071 | 1 | 1.035 |
|  |  | Covariate | HEI-2015 | 1.103 | 1 | 1.050 |
|  |  | Covariate | eGFR | 1.183 | 1 | 1.087 |
|  | ALI–ePWV joint categories | Core exposure | ALI–ePWV joint categories | 1.998 | 3 | 1.122 |
|  |  | Covariate | Age | 2.028 | 1 | 1.424 |
|  |  | Covariate | Sex | 1.316 | 1 | 1.147 |
|  |  | Covariate | Race/ethnicity | 1.328 | 3 | 1.048 |
|  |  | Covariate | Marital status | 1.266 | 2 | 1.061 |
|  |  | Covariate | Education level | 1.198 | 2 | 1.046 |
|  |  | Covariate | Smoking status | 1.385 | 2 | 1.085 |
|  |  | Covariate | Alcohol consumption | 1.298 | 2 | 1.067 |
|  |  | Covariate | Hypertension | 1.093 | 1 | 1.046 |
|  |  | Covariate | Hyperlipidemia | 1.035 | 1 | 1.017 |
|  |  | Covariate | Cancer | 1.080 | 1 | 1.039 |
|  |  | Covariate | HEI-2015 | 1.109 | 1 | 1.053 |
|  |  | Covariate | eGFR | 1.181 | 1 | 1.087 |
| Premature mortality | ALI (per 10-unit increase) | Core exposure | ALI (per 10-unit increase) | 1.130 | 1 | 1.063 |
|  |  | Covariate | Age | 1.057 | 1 | 1.028 |
|  |  | Covariate | Sex | 1.194 | 1 | 1.093 |
|  |  | Covariate | Race/ethnicity | 1.379 | 3 | 1.055 |
|  |  | Covariate | Marital status | 1.258 | 2 | 1.059 |
|  |  | Covariate | Education level | 1.175 | 2 | 1.041 |
|  |  | Covariate | Smoking status | 1.286 | 2 | 1.065 |
|  |  | Covariate | Alcohol consumption | 1.213 | 2 | 1.050 |
|  |  | Covariate | Hypertension | 1.066 | 1 | 1.032 |
|  |  | Covariate | Hyperlipidemia | 1.038 | 1 | 1.019 |
|  |  | Covariate | Cancer | 1.066 | 1 | 1.033 |
|  |  | Covariate | HEI-2015 | 1.077 | 1 | 1.038 |
|  |  | Covariate | eGFR | 1.186 | 1 | 1.089 |
|  | ePWV | Core exposure | ePWV (per 1 m/s increase) | 1.256 | 1 | 1.121 |
|  |  | Covariate | Age | 1.170 | 1 | 1.081 |
|  |  | Covariate | Sex | 1.200 | 1 | 1.095 |
|  |  | Covariate | Race/ethnicity | 1.345 | 3 | 1.051 |
|  |  | Covariate | Marital status | 1.250 | 2 | 1.057 |
|  |  | Covariate | Education level | 1.197 | 2 | 1.046 |
|  |  | Covariate | Smoking status | 1.288 | 2 | 1.065 |
|  |  | Covariate | Alcohol consumption | 1.226 | 2 | 1.052 |
|  |  | Covariate | Hypertension | 1.099 | 1 | 1.048 |
|  |  | Covariate | Hyperlipidemia | 1.049 | 1 | 1.024 |
|  |  | Covariate | Cancer | 1.061 | 1 | 1.030 |
|  |  | Covariate | HEI-2015 | 1.081 | 1 | 1.040 |
|  |  | Covariate | eGFR | 1.116 | 1 | 1.057 |
|  | ALI–ePWV joint categories | Core exposure | ALI–ePWV joint categories | 1.445 | 3 | 1.063 |
|  |  | Covariate | Age | 1.191 | 1 | 1.091 |
|  |  | Covariate | Sex | 1.215 | 1 | 1.102 |
|  |  | Covariate | Race/ethnicity | 1.372 | 3 | 1.054 |
|  |  | Covariate | Marital status | 1.269 | 2 | 1.061 |
|  |  | Covariate | Education level | 1.199 | 2 | 1.046 |
|  |  | Covariate | Smoking status | 1.324 | 2 | 1.073 |
|  |  | Covariate | Alcohol consumption | 1.244 | 2 | 1.056 |
|  |  | Covariate | Hypertension | 1.101 | 1 | 1.049 |
|  |  | Covariate | Hyperlipidemia | 1.047 | 1 | 1.023 |
|  |  | Covariate | Cancer | 1.076 | 1 | 1.037 |
|  |  | Covariate | HEI-2015 | 1.088 | 1 | 1.043 |
|  |  | Covariate | eGFR | 1.195 | 1 | 1.093 |

This detailed table reports VIF or GVIF values for the core exposure and all Model 3 covariates within each exposure-outcome model.

For categorical predictors with more than one degree of freedom, generalized variance inflation factors and GVIF-adjusted values are reported.

The primary Cox regression estimates were obtained from survey-weighted Cox models; this table is intended as a diagnostic analysis for collinearity.

Abbreviations: VIF, variance inflation factor; GVIF, generalized variance inflation factor; ALI, advanced lung cancer inflammation index; ePWV, estimated pulse wave velocity.

**Table S10.** Sensitivity analysis using alternative adjustment sets for ePWV-related models.

| **Exposure** | **Events/n** | **Original Model 3** | | **Without hypertension** | | **Without age and hypertension** | | **Without age** | |
| --- | --- | --- | --- | --- | --- | --- | --- | --- | --- |
|  |  | **HR (95%CI)** | **P-value** | **HR (95%CI)** | **P-value** | **HR (95%CI)** | **P-value** | **HR (95%CI)** | **P-value** |
| **All-cause mortality** |  |  |  |  |  |  |  |  |  |
| ePWV (per 1 m/s increase) | 911/1992 | 1.139 (1.072, 1.211) | **<0.001** | 1.132 (1.065, 1.203) | **<0.001** | 1.190 (1.128, 1.257) | **<0.001** | 1.197 (1.133, 1.264) | **<0.001** |
| **ePWV quartiles** |  |  |  |  |  |  |  |  |  |
| Q1 | 131/498 | Ref. |  | Ref. |  | Ref. |  | Ref. |  |
| Q2 | 195/498 | 1.333 (1.034, 1.719) | **0.027** | 1.319 (1.021, 1.703) | **0.034** | 1.418 (1.096, 1.835) | **0.008** | 1.434 (1.108, 1.856) | **0.006** |
| Q3 | 256/498 | 1.464 (1.050, 2.041) | **0.025** | 1.431 (1.024, 2.000) | **0.036** | 1.798 (1.331, 2.430) | **<0.001** | 1.830 (1.353, 2.476) | **<0.001** |
| Q4 | 329/498 | 1.928 (1.372, 2.711) | **<0.001** | 1.871 (1.325, 2.642) | **<0.001** | 2.468 (1.834, 3.322) | **<0.001** | 2.529 (1.877, 3.407) | **<0.001** |
| P for trend |  |  | **<0.001** |  | **<0.001** |  | **<0.001** |  | **<0.001** |
| **ALI–ePWV joint categories** |  |  |  |  |  |  |  |  |  |
| ALI <34.33 & ePWV >= 10.18 | 188/264 | Ref. |  | Ref. |  | Ref. |  | Ref. |  |
| ALI <34.33 & ePWV < 10.18 | 63/103 | 0.961 (0.644, 1.433) | 0.845 | 0.977 (0.654, 1.459) | 0.910 | 0.801 (0.546, 1.175) | 0.257 | 0.789 (0.539, 1.156) | 0.224 |
| ALI >=34.33 & ePWV >= 10.18 | 478/903 | 0.575 (0.478, 0.692) | **<0.001** | 0.577 (0.481, 0.693) | **<0.001** | 0.563 (0.467, 0.678) | **<0.001** | 0.561 (0.464, 0.677) | **<0.001** |
| ALI >=34.33 & ePWV < 10.18 | 182/722 | 0.382 (0.280, 0.520) | **<0.001** | 0.388 (0.286, 0.526) | **<0.001** | 0.308 (0.238, 0.399) | **<0.001** | 0.304 (0.234, 0.395) | **<0.001** |
| P for trend |  |  | **<0.001** |  | **<0.001** |  | **<0.001** |  | **<0.001** |
| **CVD mortality** |  |  |  |  |  |  |  |  |  |
| ePWV (per 1 m/s increase) | 368/1992 | 1.216 (1.102, 1.341) | **<0.001** | 1.205 (1.091, 1.331) | **<0.001** | 1.268 (1.161, 1.385) | **<0.001** | 1.277 (1.170, 1.393) | **<0.001** |
| **ePWV quartiles** |  |  |  |  |  |  |  |  |  |
| Q1 | 45/498 | Ref. |  | Ref. |  | Ref. |  | Ref. |  |
| Q2 | 84/498 | 1.600 (1.035, 2.472) | **0.034** | 1.587 (1.024, 2.458) | **0.039** | 1.784 (1.170, 2.720) | **0.007** | 1.800 (1.182, 2.741) | **0.006** |
| Q3 | 94/498 | 1.398 (0.796, 2.457) | 0.244 | 1.372 (0.775, 2.429) | 0.278 | 1.976 (1.213, 3.220) | **0.006** | 2.012 (1.243, 3.259) | **0.004** |
| Q4 | 145/498 | 2.037 (1.186, 3.498) | **0.010** | 1.986 (1.142, 3.454) | **0.015** | 3.079 (1.879, 5.046) | **<0.001** | 3.156 (1.941, 5.133) | **<0.001** |
| P for trend |  |  | **0.016** |  | **0.025** |  | **<0.001** |  | **<0.001** |
| **ALI–ePWV joint categories** |  |  |  |  |  |  |  |  |  |
| ALI <34.33 & ePWV >= 10.18 | 72/264 | Ref. |  | Ref. |  | Ref. |  | Ref. |  |
| ALI <34.33 & ePWV < 10.18 | 23/103 | 0.959 (0.534, 1.721) | 0.888 | 0.977 (0.546, 1.746) | 0.937 | 0.755 (0.438, 1.301) | 0.312 | 0.741 (0.430, 1.278) | 0.282 |
| ALI >=34.33 & ePWV >= 10.18 | 207/903 | 0.673 (0.490, 0.923) | **0.014** | 0.675 (0.492, 0.926) | **0.015** | 0.652 (0.473, 0.900) | **0.009** | 0.650 (0.471, 0.896) | **0.008** |
| ALI >=34.33 & ePWV < 10.18 | 66/722 | 0.379 (0.228, 0.630) | **<0.001** | 0.385 (0.233, 0.636) | **<0.001** | 0.287 (0.192, 0.430) | **<0.001** | 0.283 (0.190, 0.421) | **<0.001** |
| P for trend |  |  | **<0.001** |  | **<0.001** |  | **<0.001** |  | **<0.001** |
| **Premature mortality** |  |  |  |  |  |  |  |  |  |
| ePWV (per 1 m/s increase) | 313/1370 | 1.028 (0.918, 1.151) | 0.635 | 1.043 (0.928, 1.171) | 0.481 | 1.027 (0.919, 1.149) | 0.636 | 1.015 (0.910, 1.133) | 0.786 |
| **ePWV quartiles** |  |  |  |  |  |  |  |  |  |
| Q1 | 122/498 | Ref. |  | Ref. |  | Ref. |  | Ref. |  |
| Q2 | 121/478 | 1.166 (0.840, 1.618) | 0.358 | 1.189 (0.850, 1.662) | 0.313 | 1.169 (0.835, 1.636) | 0.363 | 1.150 (0.827, 1.599) | 0.408 |
| Q3 | 55/309 | 1.178 (0.707, 1.963) | 0.531 | 1.238 (0.738, 2.078) | 0.419 | 1.155 (0.697, 1.914) | 0.576 | 1.113 (0.676, 1.834) | 0.674 |
| Q4 | 15/85 | 0.951 (0.496, 1.824) | 0.881 | 1.022 (0.532, 1.963) | 0.949 | 0.961 (0.490, 1.881) | 0.906 | 0.902 (0.463, 1.759) | 0.763 |
| P for trend |  |  | 0.526 |  | 0.385 |  | 0.566 |  | 0.705 |
| **ALI–ePWV joint categories** |  |  |  |  |  |  |  |  |  |
| ALI <34.33 & ePWV >= 10.18 | 19/77 | Ref. |  | Ref. |  | Ref. |  | Ref. |  |
| ALI <34.33 & ePWV < 10.18 | 53/103 | 1.087 (0.624, 1.896) | 0.768 | 1.060 (0.612, 1.837) | 0.836 | 1.188 (0.700, 2.015) | 0.524 | 1.200 (0.701, 2.055) | 0.507 |
| ALI >=34.33 & ePWV >= 10.18 | 90/473 | 0.500 (0.295, 0.847) | **0.010** | 0.506 (0.300, 0.853) | **0.011** | 0.535 (0.314, 0.911) | **0.021** | 0.524 (0.306, 0.900) | **0.019** |
| ALI >=34.33 & ePWV < 10.18 | 151/717 | 0.402 (0.245, 0.659) | **<0.001** | 0.389 (0.236, 0.640) | **<0.001** | 0.435 (0.268, 0.704) | **<0.001** | 0.442 (0.273, 0.718) | **<0.001** |
| P for trend |  |  | **<0.001** |  | **<0.001** |  | **<0.001** |  | **<0.001** |

This analysis evaluated whether the ePWV-related associations were sensitive to adjustment for age and hypertension, because ePWV is derived from age and blood pressure.

The original Model 3 adjusted for age, sex, race/ethnicity, marital status, education level, smoking status, alcohol consumption, hypertension, hyperlipidemia, cancer, HEI-2015 and eGFR.

Events/n are presented as the number of events divided by the number of participants in each exposure category.

For premature mortality, analyses were restricted to participants younger than 75 years at baseline and follow-up was censored at 75 years of age.

Abbreviations: Ref, reference; CVD, cardiovascular disease; ALI, advanced lung cancer inflammation index; ePWV, estimated pulse wave velocity; HR, hazard ratio; CI, confidence interval.

**Table S11.** Detailed proportional hazards assumption tests for the fully adjusted Cox models.

| **Outcome** | **Exposure model** | **Term role** | **Term** | **PH test Chisq** | **PH test Df** | **PH test P-value** |
| --- | --- | --- | --- | --- | --- | --- |
| All-cause mortality | ALI (per 10-unit increase) | Core exposure | ALI (per 10-unit increase) | 22.037 | 1 | **<0.001** |
|  |  | Covariate | Age | 1.404 | 1 | 0.236 |
|  |  | Covariate | Sex | 0.049 | 1 | 0.825 |
|  |  | Covariate | Race/ethnicity | 4.006 | 3 | 0.261 |
|  |  | Covariate | Marital status | 1.583 | 2 | 0.453 |
|  |  | Covariate | Education level | 0.785 | 2 | 0.675 |
|  |  | Covariate | Smoking status | 1.050 | 2 | 0.592 |
|  |  | Covariate | Alcohol consumption | 2.998 | 2 | 0.223 |
|  |  | Covariate | Hypertension | 0.036 | 1 | 0.849 |
|  |  | Covariate | Hyperlipidemia | 2.644 | 1 | 0.104 |
|  |  | Covariate | Cancer | 1.378 | 1 | 0.240 |
|  |  | Covariate | HEI-2015 | 1.851 | 1 | 0.174 |
|  |  | Covariate | eGFR | 1.476 | 1 | 0.224 |
|  |  | Global test | Global test | 46.178 | 19 | **<0.001** |
|  | ePWV | Core exposure | ePWV (per 1 m/s increase) | 5.319 | 1 | **0.021** |
|  |  | Covariate | Age | 1.428 | 1 | 0.232 |
|  |  | Covariate | Sex | 0.058 | 1 | 0.809 |
|  |  | Covariate | Race/ethnicity | 4.638 | 3 | 0.200 |
|  |  | Covariate | Marital status | 2.507 | 2 | 0.285 |
|  |  | Covariate | Education level | 0.368 | 2 | 0.832 |
|  |  | Covariate | Smoking status | 1.585 | 2 | 0.453 |
|  |  | Covariate | Alcohol consumption | 4.368 | 2 | 0.113 |
|  |  | Covariate | Hypertension | 0.063 | 1 | 0.802 |
|  |  | Covariate | Hyperlipidemia | 2.566 | 1 | 0.109 |
|  |  | Covariate | Cancer | 1.809 | 1 | 0.179 |
|  |  | Covariate | HEI-2015 | 1.263 | 1 | 0.261 |
|  |  | Covariate | eGFR | 2.205 | 1 | 0.138 |
|  |  | Global test | Global test | 31.413 | 19 | **0.036** |
|  | ALI–ePWV joint categories | Core exposure | ALI–ePWV joint categories | 11.051 | 3 | **0.011** |
|  |  | Covariate | Age | 1.327 | 1 | 0.249 |
|  |  | Covariate | Sex | 0.118 | 1 | 0.731 |
|  |  | Covariate | Race/ethnicity | 4.531 | 3 | 0.210 |
|  |  | Covariate | Marital status | 1.935 | 2 | 0.380 |
|  |  | Covariate | Education level | 0.644 | 2 | 0.725 |
|  |  | Covariate | Smoking status | 1.093 | 2 | 0.579 |
|  |  | Covariate | Alcohol consumption | 3.233 | 2 | 0.199 |
|  |  | Covariate | Hypertension | 0.303 | 1 | 0.582 |
|  |  | Covariate | Hyperlipidemia | 2.688 | 1 | 0.101 |
|  |  | Covariate | Cancer | 1.631 | 1 | 0.202 |
|  |  | Covariate | HEI-2015 | 1.253 | 1 | 0.263 |
|  |  | Covariate | eGFR | 1.152 | 1 | 0.283 |
|  |  | Global test | Global test | 33.853 | 21 | **0.038** |
| CVD mortality | ALI (per 10-unit increase) | Core exposure | ALI (per 10-unit increase) | 4.869 | 1 | **0.027** |
|  |  | Covariate | Age | 1.119 | 1 | 0.290 |
|  |  | Covariate | Sex | 0.008 | 1 | 0.928 |
|  |  | Covariate | Race/ethnicity | 6.223 | 3 | 0.101 |
|  |  | Covariate | Marital status | 1.108 | 2 | 0.575 |
|  |  | Covariate | Education level | 0.351 | 2 | 0.839 |
|  |  | Covariate | Smoking status | 0.237 | 2 | 0.888 |
|  |  | Covariate | Alcohol consumption | 2.153 | 2 | 0.341 |
|  |  | Covariate | Hypertension | 1.646 | 1 | 0.200 |
|  |  | Covariate | Hyperlipidemia | 1.386 | 1 | 0.239 |
|  |  | Covariate | Cancer | 0.224 | 1 | 0.636 |
|  |  | Covariate | HEI-2015 | 1.327 | 1 | 0.249 |
|  |  | Covariate | eGFR | 7.141 | 1 | **0.008** |
|  |  | Global test | Global test | 31.257 | 19 | **0.038** |
|  | ePWV | Core exposure | ePWV (per 1 m/s increase) | 4.630 | 1 | **0.031** |
|  |  | Covariate | Age | 1.398 | 1 | 0.237 |
|  |  | Covariate | Sex | 0.008 | 1 | 0.931 |
|  |  | Covariate | Race/ethnicity | 6.290 | 3 | 0.098 |
|  |  | Covariate | Marital status | 1.552 | 2 | 0.460 |
|  |  | Covariate | Education level | 0.179 | 2 | 0.914 |
|  |  | Covariate | Smoking status | 0.177 | 2 | 0.915 |
|  |  | Covariate | Alcohol consumption | 2.925 | 2 | 0.232 |
|  |  | Covariate | Hypertension | 1.505 | 1 | 0.220 |
|  |  | Covariate | Hyperlipidemia | 1.472 | 1 | 0.225 |
|  |  | Covariate | Cancer | 0.139 | 1 | 0.710 |
|  |  | Covariate | HEI-2015 | 1.174 | 1 | 0.278 |
|  |  | Covariate | eGFR | 7.707 | 1 | **0.006** |
|  |  | Global test | Global test | 32.778 | 19 | **0.025** |
|  | ALI–ePWV joint categories | Core exposure | ALI–ePWV joint categories | 4.674 | 3 | 0.197 |
|  |  | Covariate | Age | 0.965 | 1 | 0.326 |
|  |  | Covariate | Sex | 0.027 | 1 | 0.869 |
|  |  | Covariate | Race/ethnicity | 6.017 | 3 | 0.111 |
|  |  | Covariate | Marital status | 1.218 | 2 | 0.544 |
|  |  | Covariate | Education level | 0.363 | 2 | 0.834 |
|  |  | Covariate | Smoking status | 0.229 | 2 | 0.892 |
|  |  | Covariate | Alcohol consumption | 2.098 | 2 | 0.350 |
|  |  | Covariate | Hypertension | 1.194 | 1 | 0.275 |
|  |  | Covariate | Hyperlipidemia | 1.552 | 1 | 0.213 |
|  |  | Covariate | Cancer | 0.194 | 1 | 0.659 |
|  |  | Covariate | HEI-2015 | 1.069 | 1 | 0.301 |
|  |  | Covariate | eGFR | 7.098 | 1 | **0.008** |
|  |  | Global test | Global test | 30.832 | 21 | 0.077 |
| Premature mortality | ALI (per 10-unit increase) | Core exposure | ALI (per 10-unit increase) | 9.063 | 1 | **0.003** |
|  |  | Covariate | Age | 0.131 | 1 | 0.717 |
|  |  | Covariate | Sex | 0.240 | 1 | 0.624 |
|  |  | Covariate | Race/ethnicity | 0.118 | 3 | 0.990 |
|  |  | Covariate | Marital status | 1.595 | 2 | 0.450 |
|  |  | Covariate | Education level | 0.618 | 2 | 0.734 |
|  |  | Covariate | Smoking status | 4.416 | 2 | 0.110 |
|  |  | Covariate | Alcohol consumption | 4.932 | 2 | 0.085 |
|  |  | Covariate | Hypertension | 0.540 | 1 | 0.463 |
|  |  | Covariate | Hyperlipidemia | 3.495 | 1 | 0.062 |
|  |  | Covariate | Cancer | 3.658 | 1 | 0.056 |
|  |  | Covariate | HEI-2015 | 4.505 | 1 | **0.034** |
|  |  | Covariate | eGFR | 0.830 | 1 | 0.362 |
|  |  | Global test | Global test | 38.424 | 19 | **0.005** |
|  | ePWV | Core exposure | ePWV (per 1 m/s increase) | 1.760 | 1 | 0.185 |
|  |  | Covariate | Age | 0.129 | 1 | 0.719 |
|  |  | Covariate | Sex | 0.213 | 1 | 0.645 |
|  |  | Covariate | Race/ethnicity | 0.185 | 3 | 0.980 |
|  |  | Covariate | Marital status | 1.698 | 2 | 0.428 |
|  |  | Covariate | Education level | 0.433 | 2 | 0.805 |
|  |  | Covariate | Smoking status | 4.453 | 2 | 0.108 |
|  |  | Covariate | Alcohol consumption | 5.224 | 2 | 0.073 |
|  |  | Covariate | Hypertension | 0.535 | 1 | 0.464 |
|  |  | Covariate | Hyperlipidemia | 3.315 | 1 | 0.069 |
|  |  | Covariate | Cancer | 4.256 | 1 | **0.039** |
|  |  | Covariate | HEI-2015 | 4.344 | 1 | **0.037** |
|  |  | Covariate | eGFR | 0.735 | 1 | 0.391 |
|  |  | Global test | Global test | 32.824 | 19 | **0.025** |
|  | ALI–ePWV joint categories | Core exposure | ALI–ePWV joint categories | 3.849 | 3 | 0.278 |
|  |  | Covariate | Age | 0.082 | 1 | 0.774 |
|  |  | Covariate | Sex | 0.140 | 1 | 0.708 |
|  |  | Covariate | Race/ethnicity | 0.269 | 3 | 0.966 |
|  |  | Covariate | Marital status | 1.384 | 2 | 0.501 |
|  |  | Covariate | Education level | 0.601 | 2 | 0.740 |
|  |  | Covariate | Smoking status | 4.042 | 2 | 0.133 |
|  |  | Covariate | Alcohol consumption | 4.772 | 2 | 0.092 |
|  |  | Covariate | Hypertension | 0.249 | 1 | 0.618 |
|  |  | Covariate | Hyperlipidemia | 3.512 | 1 | 0.061 |
|  |  | Covariate | Cancer | 3.351 | 1 | 0.067 |
|  |  | Covariate | HEI-2015 | 4.827 | 1 | **0.028** |
|  |  | Covariate | eGFR | 0.855 | 1 | 0.355 |
|  |  | Global test | Global test | 33.475 | 21 | **0.041** |

This detailed table reports Schoenfeld residual-based PH tests for the core exposure term, each Model 3 covariate, and the global test within each exposure-outcome model.

PH diagnostics were performed using Cox models with the same covariate set as the fully adjusted Model 3.

A P-value <0.05 suggests potential non-proportionality and should be interpreted as a model diagnostic rather than as an effect estimate.

Abbreviations: PH, proportional hazards; ALI, advanced lung cancer inflammation index; ePWV, estimated pulse wave velocity.

**Table S12.** Accelerated failure time model sensitivity analysis for the core exposure-mortality associations.

| **Outcome** | **Exposure model** | **Exposure** | **Events/n** | **Selected AFT distribution** | **AFT fitting method** | **Time ratio (95%CI)** | **P-value** |
| --- | --- | --- | --- | --- | --- | --- | --- |
| All-cause mortality | ALI (per 10-unit increase) | ALI (per 10-unit increase) | 911/1992 | weibull | weighted robust clustered | 1.071 (1.067, 1.075) | **<0.001** |
|  | ALI quartiles | Q1 | 319/498 |  |  | Ref. |  |
|  |  | Q2 | 232/498 |  |  | 1.306 (1.234, 1.383) | **<0.001** |
|  |  | Q3 | 206/498 |  |  | 1.399 (1.260, 1.554) | **<0.001** |
|  |  | Q4 | 154/498 |  |  | 1.638 (1.589, 1.688) | **<0.001** |
|  |  | P for trend |  |  |  |  | **<0.001** |
|  | ePWV (per 1 m/s increase) | ePWV (per 1 m/s increase) | 911/1992 |  |  | 0.919 (0.903, 0.936) | **<0.001** |
|  | ePWV quartiles | Q1 | 131/498 |  |  | Ref. |  |
|  |  | Q2 | 195/498 |  |  | 0.835 (0.793, 0.879) | **<0.001** |
|  |  | Q3 | 256/498 |  |  | 0.783 (0.741, 0.828) | **<0.001** |
|  |  | Q4 | 329/498 |  |  | 0.655 (0.633, 0.677) | **<0.001** |
|  |  | P for trend |  |  |  |  | **<0.001** |
|  | ALI–ePWV joint categories | ALI <34.33 & ePWV >= 10.18 | 188/264 |  |  | Ref. |  |
|  |  | ALI <34.33 & ePWV < 10.18 | 63/103 |  |  | 1.028 (0.994, 1.064) | 0.109 |
|  |  | ALI >=34.33 & ePWV >= 10.18 | 478/903 |  |  | 1.428 (1.362, 1.496) | **<0.001** |
|  |  | ALI >=34.33 & ePWV < 10.18 | 182/722 |  |  | 1.860 (1.736, 1.993) | **<0.001** |
|  |  | P for trend |  |  |  |  | **<0.001** |
| CVD mortality | ALI (per 10-unit increase) | ALI (per 10-unit increase) | 368/1992 | loglogistic |  | 1.050 (1.032, 1.069) | **<0.001** |
|  | ALI quartiles | Q1 | 119/498 |  |  | Ref. |  |
|  |  | Q2 | 89/498 |  |  | 1.362 (1.312, 1.413) | **<0.001** |
|  |  | Q3 | 95/498 |  |  | 1.179 (1.104, 1.260) | **<0.001** |
|  |  | Q4 | 65/498 |  |  | 1.520 (1.336, 1.728) | **<0.001** |
|  |  | P for trend |  |  |  |  | **<0.001** |
|  | ePWV (per 1 m/s increase) | ePWV (per 1 m/s increase) | 368/1992 |  |  | 0.880 (0.876, 0.884) | **<0.001** |
|  | ePWV quartiles | Q1 | 45/498 |  |  | Ref. |  |
|  |  | Q2 | 84/498 |  |  | 0.746 (0.594, 0.936) | **0.012** |
|  |  | Q3 | 94/498 |  |  | 0.801 (0.580, 1.106) | 0.178 |
|  |  | Q4 | 145/498 |  |  | 0.609 (0.440, 0.844) | **0.003** |
|  |  | P for trend |  |  |  |  | **<0.001** |
|  | ALI–ePWV joint categories | ALI <34.33 & ePWV >= 10.18 | 72/264 |  |  | Ref. |  |
|  |  | ALI <34.33 & ePWV < 10.18 | 23/103 |  |  | 1.073 (0.978, 1.179) | 0.138 |
|  |  | ALI >=34.33 & ePWV >= 10.18 | 207/903 |  |  | 1.354 (1.097, 1.672) | **0.005** |
|  |  | ALI >=34.33 & ePWV < 10.18 | 66/722 |  |  | 1.949 (1.793, 2.119) | **<0.001** |
|  |  | P for trend |  |  |  |  | **<0.001** |
| Premature mortality | ALI (per 10-unit increase) | ALI (per 10-unit increase) | 313/1370 | weibull |  | 1.088 (1.076, 1.100) | **<0.001** |
|  | ALI quartiles | Q1 | 96/257 |  |  | Ref. |  |
|  |  | Q2 | 77/340 |  |  | 1.451 (1.237, 1.702) | **<0.001** |
|  |  | Q3 | 73/357 |  |  | 1.490 (1.383, 1.605) | **<0.001** |
|  |  | Q4 | 67/416 |  |  | 1.934 (1.776, 2.107) | **<0.001** |
|  |  | P for trend |  |  |  |  | **<0.001** |
|  | ePWV (per 1 m/s increase) | ePWV (per 1 m/s increase) | 313/1370 |  |  | 0.992 (0.967, 1.018) | 0.540 |
|  | ePWV quartiles | Q1 | 122/498 |  |  | Ref. |  |
|  |  | Q2 | 121/478 |  |  | 0.920 (0.888, 0.953) | **<0.001** |
|  |  | Q3 | 55/309 |  |  | 0.917 (0.746, 1.127) | 0.410 |
|  |  | Q4 | 15/85 |  |  | 1.099 (0.687, 1.760) | 0.693 |
|  |  | P for trend |  |  |  |  | **<0.001** |
|  | ALI–ePWV joint categories | ALI <34.33 & ePWV >= 10.18 | 19/77 |  |  | Ref. |  |
|  |  | ALI <34.33 & ePWV < 10.18 | 53/103 |  |  | 0.939 (0.865, 1.020) | 0.136 |
|  |  | ALI >=34.33 & ePWV >= 10.18 | 90/473 |  |  | 1.660 (1.556, 1.770) | **<0.001** |
|  |  | ALI >=34.33 & ePWV < 10.18 | 151/717 |  |  | 1.866 (1.726, 2.018) | **<0.001** |
|  |  | P for trend |  |  |  |  | **<0.001** |

Parametric accelerated failure time models were fitted as sensitivity analyses because some Cox models showed potential non-proportionality in Schoenfeld residual diagnostics.

The candidate AFT distributions were log-normal, log-logistic and Weibull; the distribution with the lowest AIC was selected for each outcome-exposure model.

Models preferentially used normalized NHANES examination weights with robust variance and primary-sampling-unit clustering; if convergence failed, the fitting procedure automatically used the most stable lower-complexity alternative, as reported in the table.

A time ratio greater than 1 indicates longer survival time, whereas a time ratio less than 1 indicates shorter survival time.

The same covariate set as Model 3 was used.

For premature mortality, analyses were restricted to participants younger than 75 years at baseline and follow-up was censored at 75 years of age.

Abbreviations: AFT, accelerated failure time; ALI, advanced lung cancer inflammation index; ePWV, estimated pulse wave velocity; CVD, cardiovascular disease; CI, confidence interval.

**Table S13.** Competing-risk sensitivity analysis for cardiovascular mortality.

| **Exposure** | **Events/n** | **Subdistribution HR (95%CI)** | **P-value** |
| --- | --- | --- | --- |
| **CVD mortality** |  |  |  |
| ALI (per 10-unit increase) | 368/1992 | 0.973 (0.932, 1.016) | 0.209 |
| **ALI quartiles** |  |  |  |
| Q1 | 119/498 | Ref. |  |
| Q2 | 89/498 | 0.831 (0.623, 1.107) | 0.206 |
| Q3 | 95/498 | 0.963 (0.725, 1.278) | 0.793 |
| Q4 | 65/498 | 0.766 (0.552, 1.061) | 0.109 |
| P for trend |  |  | 0.230 |
| ePWV (per 1 m/s increase) | 368/1992 | 1.197 (1.103, 1.299) | **<0.001** |
| **ePWV quartiles** |  |  |  |
| Q1 | 45/498 | Ref. |  |
| Q2 | 84/498 | 1.555 (1.062, 2.276) | **0.023** |
| Q3 | 94/498 | 1.293 (0.819, 2.043) | 0.270 |
| Q4 | 145/498 | 1.981 (1.230, 3.190) | **0.005** |
| P for trend |  |  | **0.005** |
| **ALI–ePWV joint categories** |  |  |  |
| ALI <34.33 & ePWV >= 10.18 | 72/264 | Ref. |  |
| ALI <34.33 & ePWV < 10.18 | 23/103 | 1.125 (0.657, 1.928) | 0.667 |
| ALI >=34.33 & ePWV >= 10.18 | 207/903 | 0.918 (0.686, 1.227) | 0.561 |
| ALI >=34.33 & ePWV < 10.18 | 66/722 | 0.528 (0.343, 0.815) | **0.004** |
| P for trend |  |  | **0.014** |

Fine-Gray competing-risk models were used for cardiovascular mortality, treating non-cardiovascular death as a competing event.

The same covariates as Model 3 were included in the competing-risk models.

This analysis was performed as a supplementary competing-risk assessment and should be interpreted as a sensitivity analysis rather than a replacement for the primary survey-weighted Cox models.

Abbreviations: Ref, reference; ALI, advanced lung cancer inflammation index; ePWV, estimated pulse wave velocity; CVD, cardiovascular disease; HR, hazard ratio; CI, confidence interval.
